# The Roles of Cardiovascular H_2_-Histamine Receptors Under Normal and Pathophysiological Conditions

**DOI:** 10.3389/fphar.2021.732842

**Published:** 2021-12-20

**Authors:** Joachim Neumann, Uwe Kirchhefer, Stefan Dhein, Britt Hofmann, Ulrich Gergs

**Affiliations:** ^1^ Institut für Pharmakologie und Toxikologie, Medizinische Fakultät, Martin-Luther-Universität Halle-Wittenberg, Halle, Germany; ^2^ Institut für Pharmakologie und Toxikologie, Westfälische Wilhelms-Universität, Münster, Germany; ^3^ Landratsamt Altenburger Land, Altenburg, Germany; ^4^ Herzchirurgie, Medizinische Fakultät, Martin-Luther-Universität Halle-Wittenberg, Halle, Germany

**Keywords:** H2 histamine receptor, contractil effect, ischemia - reperfusion, arrhythmias, heart failure

## Abstract

This review addresses pharmacological, structural and functional relationships among H_2_-histamine receptors and H_1_-histamine receptors in the mammalian heart. The role of both receptors in the regulation of force and rhythm, including their electrophysiological effects on the mammalian heart, will then be discussed in context. The potential clinical role of cardiac H_2_-histamine-receptors in cardiac diseases will be examined. The use of H_2_-histamine receptor agonists to acutely increase the force of contraction will be discussed. Special attention will be paid to the potential role of cardiac H_2_-histamine receptors in the genesis of cardiac arrhythmias. Moreover, novel findings on the putative role of H_2_-histamine receptor antagonists in treating chronic heart failure in animal models and patients will be reviewed. Some limitations in our biochemical understanding of the cardiac role of H_2_-histamine receptors will be discussed. Recommendations for further basic and translational research on cardiac H_2_-histamine receptors will be offered. We will speculate whether new knowledge might lead to novel roles of H_2_-histamine receptors in cardiac disease and whether cardiomyocyte specific H_2_-histamine receptor agonists and antagonists should be developed.

## 1 Introduction

Although many reviews on histamine receptors have been published (Marone et al., 2014; Panula et al., 2015; Marino and Levi 2018; Zhang et al., 2018), few up-to-date reviews have focused on cardiac histamine receptors. Moreover, the most recent review was published by Hattori et al., in 2017. The present work reviews the most recent works on this topic in the relevant literature.

The “histamine” molecule was named by Fühner (1912) based on its chemical structure, which is β-imidazolyl-amin(e). The term histamine was derived from the Greek words for tissue (“histos” or΄ΙΣΤΟΣ) and “amine” (a nitrogen containing alkyl-derivate), which translate as the amine in the tissue. Histamine was first synthesised by two chemists from Freiburg im Breisgau using a battery of structurally similar compounds (Windaus and Vogt 1907) without studying their presence or function in animals. Later, Ackermann (also in Freiburg, Germany) found that bacteria could produce histamine from histidine, proving that histamine could be produced in nature and not only in the test tube (Ackermann 1910; Ackermann and Kutscher 1910). Previously, histamine was shown to increase the cardiac force of contraction, to increase the beating rate of the heart and to induce arrhythmias. Indeed, in early studies, synthetic histamine was found to exert a positive inotropic effect (PIE) and a positive chronotropic effect (PCE) in isolated perfused hearts (Ackermann and Kutscher 1910; Dale and Laidlaw 1910, 1911; Einis 1913). Histamine-induced arrhythmias were also reported in these early papers. For example, histamine led to asystole or third-degree atrioventricular block in isolated buffer-perfused spontaneously beating frog hearts (Einis 1913).

Over time, interest in cardiac histamine receptors has varied, depending on the development of new methods. In the early years (1910–1930), whole animal experiments were predominant. Then H_1_-histamine receptor (H_1_R) antagonists became available, which were used to treat anaphylactic shock. From the 1950s to 1980, electrophysiological experiments in multicellular cardiac preparations and then on isolated cardiomyocytes in animals and humans became feasible, and they were used in cardiac histamine research. In 1972, a paper on H_2_-histamine receptor (H_2_R) antagonists was published (Black et al., 1972). These H_2_R antagonists were quickly used to differentiate between H_1_R- and H_2_R-mediated cardiac functions. H_2_R antagonists were used in whole animal experiments, in experiments using atrial or ventricular multicellular preparations, and in isolated muscle cell studies. From 1970 to 1980, signal transductions of histamine receptors were studied using biochemical methods (adenylyl cyclase, 3′,5′-cyclic adenosine monophosphate [cAMP], and inositol trisphosphate [IP_3_] measurements). When histamine receptors were cloned in the 1990s, molecular studies and mutational studies on H_1_- and H_2_-histamine receptors became feasible. The next steps were performed in genetic studies using adenoviral constructs or in studies on mice using gene deletion methods and gene overexpression methods to examined H_2_-histamine receptors. Around 1980, a surge in clinical studies on H_2_R agonists appeared, which did not lead to clinical application because of side effects. The use of these H_2_R agonists could have been impaired by side effects such as acid production in the stomach (Felix et al., 1991a, 1995) or the assumption that all cAMP-increasing agents induce cardiac arrhythmias. At that time, a seminal paper was published showing that cAMP-increasing agents such as the phosphodiesterase III inhibitor milrinone or β-adrenoceptor agonists increased the ejection fraction of the left cardiac ventricle of patients with severe chronic heart failure. However, more patients died in the milrinone group than in the control group, mainly of fatal arrhythmias (Packer et al., 1991). Tariq and Aronow (2015) published a review on several cAMP-increasing agents in patients. Subsequently, the use of H_2_R antagonists has been the subject of clinical studies and trials aimed at changing the therapy for heart failure.

**FIGURE 1:**
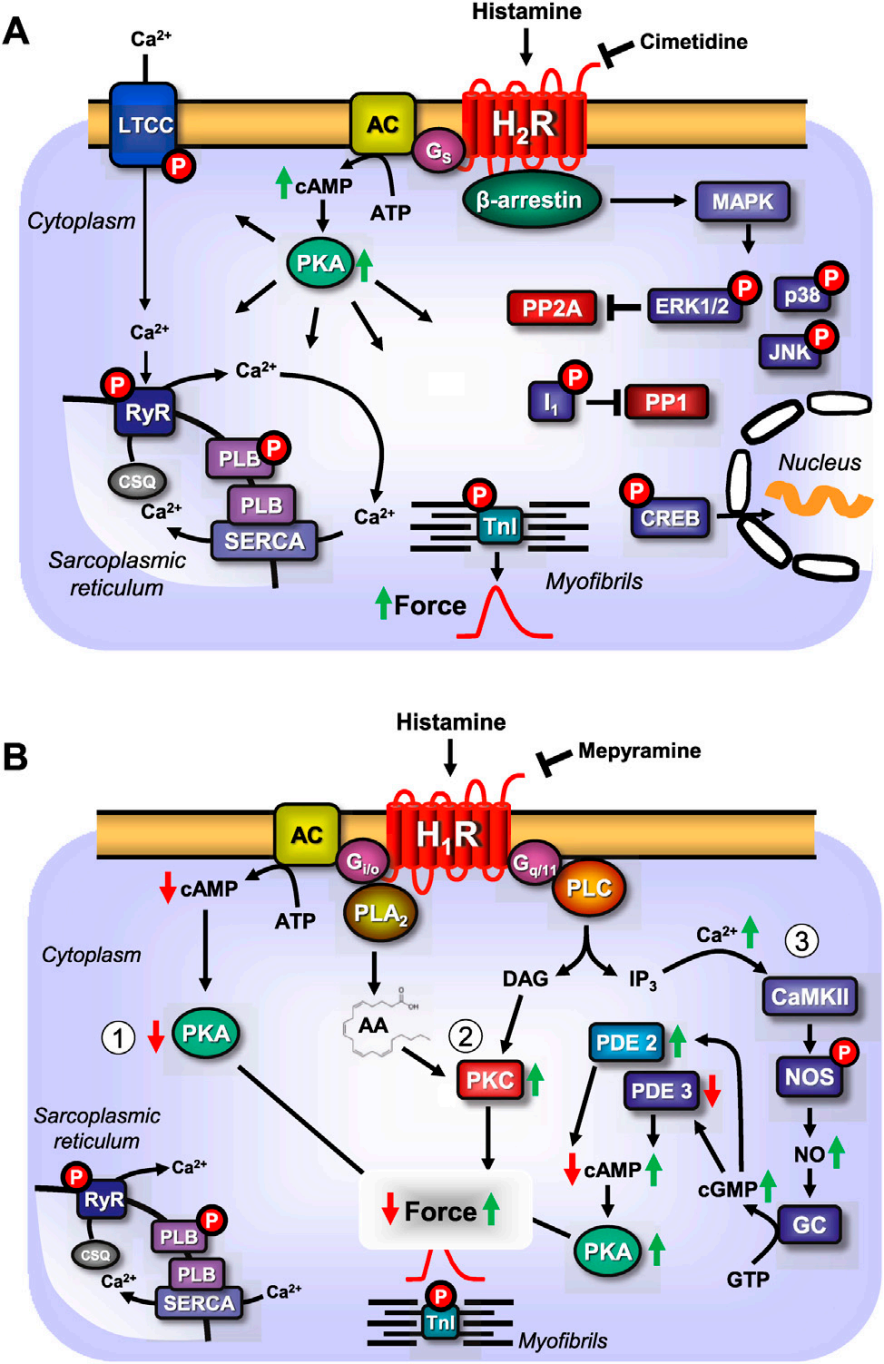
**(A)** Scheme: putative mechanism(s) of signal transduction of cardiac H_2_-histamine receptors stimulated by histamine and antagonized by cimetidine. H_2_-histamine receptors (H_2_R) can activate adenylyl cyclases (AC) via stimulatory GTP binding proteins (G_s_), which would enhance the 3′, 5′-cyclic adenosine-phosphate (cAMP)-levels in central compartments of the cardiomyocyte. This cAMP can activate cAMP-dependent protein kinase (PKA), which would increase the phosphorylation state and thereby, the activity of several regulatory proteins in the cardiomyocyte. For instance, PKA-stimulated phosphorylation increases the current through the L-type Ca^2+^ channel (LTCC) and/or the release of Ca^2+^ from the sarcoplasmic reticulum (SR) via the cardiac ryanodine receptor (RYR). This process is thought to initiate cardiac contraction. In diastole, Ca^2+^ is pumped via the SR-Ca^2+^-ATPase (SERCA) from the cytosol into the SR. Activity of SERCA is increased when PKA phosphorylates phospholamban (PLB). PKA also phosphorylates the inhibitory subunit of troponin (TnI). The phosphorylation of TnI reduces the sensitivity of the myofilaments for Ca^2+^ and thus the muscle will relax faster in diastole. The latter effect might also follow from inhibition of PP2A (a serine/threonine phosphatase: PP) activity by MAP kinases (mitogen activated protein kinases) and subsequent increased phosphorylation state and thus activation of I-1 (a specific inhibitory protein of PP1 [serine threonine protein phosphatase 1]), which will lead to decreased activity of PP1. PKA can also phosphorylate and thus activate the cAMP-dependent transcription factor (CREB). Alternatively (sometimes called the non-canonical pathway) the phosphorylation state and thus the activity of ERK1/2, JNK (c-jun N terminal kinase), p38 (p38 mitogen activated protein kinase) could be enhanced by pathways acting via arrestins. In the human heart, via H_2_-histamine receptor, cAMP-content is increased, PKA is activated, phospholamban and troponin I phosphorylation is enhanced and the open probability of the LTCC is augmented. **(B) **Scheme: putative mechanism(s) of signal transduction of cardiac H_1_-histamine-receptors, stimulated after endogenous agonist binding (histamine) on the receptor which can be abrogated by an exogenous antagonist like mepyramine. Three putative pathways are indicated with Arabic numbers. H_1_-histamine receptors (H_1_R) via (labeled 1 in the scheme) the α-subunits of the inhibitory GTP-binding proteins (Giα) can inhibit the activity of adenylyl cyclases (AC) which would reduce the 3′-5′cyclic adenosine-phosphate (cAMP)-levels in central compartments of the cardiomyocyte and thus diminish the activity of cAMP-dependent protein kinases (PKA), which eventually leads to a decline in the phosphorylation state of regulatory proteins in the cell. Alternatively (labeled 2 in the scheme) the activity of phospholipase A2 (PLA_2_) might be increased leading to formation of arachidonic acid (AA) and finally activation of protein kinase C (PKC) leading to protein phosphorylation and hence increased force generation. Lastly (labeled 3 in the scheme), H_1_-histamine-receptors may via GTP binding proteins called G_q_ or G_11_ activate phospholipase C (PLC). This would increase the level of diacylglycerol (=DAG) in the cells and thus elevate the activity of PKC. In addition, PLC leads to the formation of inositol trisphosphate (IP_3_), which can release Ca^2+^ from storage sites like the sarcoplasmic reticulum (SR), where it binds to calsequestrin (CSQ) is taken up by SR-Ca^2+^ATPAse (SERCA) which is activated when phospholamban (PLB) is phosphorylated by PKA or CaMKII. Ryanodine receptor upon their phosphorylation release Ca^2+^ from the SR which then contributes to force generation. An elevation of cytosolic Ca^2+^ is expected to bind to calmodulin and this can activate a kinase (CaMKII). This kinase can phosphorylate and activate nitric oxide (NO) synthase (NOS). This activation would lead to the enhanced formation of NO which stimulates guanylyl cyclase (GC) thus increases 3′-5′cyclic guanosine-phosphate (cGMP) levels. Elevated cGMP can reduce the activity of phosphodiesterase III (PDE III) or enhance the activity of phosphodiesterase II (PDE II). This would elevate or reduce cAMP, respectively, which would activate or inhibit PKA and eventually increase or decrease force generation. In the human heart, H_1_-histamine receptor stimulation increases cGMP- and cAMP-levels, activate PKA and increase force of contraction (Sanders et al., 1996). In contrast, others reported a decrease of force, at least in some patients after H_1_-histamine receptor stimulation (Guo et al., 1984; Du et al., 1993).

In the present review study, we distinguish four histamine receptors that, based on their chronology of detection, are called H_1_-, H_2_-, H_3_- and H_4_-histamine receptors. They belong to the large family of heptahelical receptors that are thought to be located mainly in the sarcolemma. These histamine receptors couple via at least two pathways to elicit cardiac effects. First, the histamine receptors act via well-described guanosine-tri-phosphate (GTP)-binding proteins (G-proteins). Second, as shown in Figure 1A,B, the histamine receptors use β-arrestins to couple to intracellular signal transduction pathways (Hill et al., 1997; Seifert et al., 2013; Panula et al., 2015). All four histamine receptors are present in the mammalian heart (Panula et al., 2015; Hattori et al., 2017). However, only H_1_- and H_2_-histamine receptors couple directly to force contraction or beating rate in the mammalian heart (Hattori et al., 2017). In contrast to H_3_- and H_4_-histamine receptors, H_1_- and H_2_-histamine receptors are located on the cardiomyocyte (Hattori et al., 2017). The present review study focuses on H_2_-histamine receptors because they are relevant for the positive inotropic and positive chronotropic effects of histamine in the human heart. There is still controversy in the field about whether H_1_-histamine receptors increase or decrease the force of contraction in the human heart (Guo et al., 1984; Sanders et al., 1996). However, there is agreement that H_1_-histamine receptors probably slow the intrinsic heart rate and the propagation of the heartbeat via the conduction system in the mammalian heart, including the human heart (Hattori et al., 2017). H_3_- and H_4_-histamine receptors are present on neuronal cell structures in the mammalian heart, but not on cardiomyocytes. H_3_- and H_4_-histamine receptors can inhibit the release of noradrenaline (NE) from storage sites (ganglia) in the human heart (Hattori et al., 2017).

The human H_2_R consists of 359 amino acids (Gantz et al., 1991a, 1991b; Panula et al., 2015) and is located on chromosome 5 (Hill et al., 1997; Dy and Schneider 2004; Jutel et al., 2009). There are pharmacological and genetic tools to study H_2_-histamine receptors in the heart. Genetic tools for studying the H_2_R in more detail include a strain of general, constitutive knockout (KO = deletion of a gene in a mouse) mice for H_2_R, a floxed H_2_R mouse and one mouse line with cardiac specific overexpression of H_2_R (Kobayashi et al., 2000; Gergs et al., 2019; Meng et al., 2021). Genetically modified mice with a floxed H_2_R gene (Meng et al., 2021) can be used to generate cell-specific removal or at least reduce the expression of H_2_R. Floxed mice have recently been used to delete H_2_R in endothelial cells (EC) (Meng et al., 2021). Theoretically, the floxed mouse could be used for genetic deletion of H_2_R in adult mouse cardiomyocytes. However, this experiment is not expected to be useful to reveal the exact function of H_2_R in adult cardiomyocytes in patients because adult mouse cardiomyocytes do not express functional H_2_R (Gergs et al., 2019). In adult mouse cardiomyocytes, histamine does not increase the mechanical function of the cell, and histamine has no positive inotropic effect on wild-type mouse hearts (Gergs et al., 2019). Hence, the deletion of the H_2_R in adult mouse heart or adult mouse cardiomyocytes is not likely to reveal any new information. Please note that we specify adult mouse cardiomyocytes, as foetal mouse cardiomyocytes might respond to histamine by an increase in contractility, which, to the best of our knowledge, has not yet been studied. The contractile effect of histamine in the mammalian heart is clearly age dependent, but it differs in different parts or regions of the mammalian heart, and it is species dependent (see also *Histamine and cAMP in the Heart: Age- and Species-Dependent Presence of Cardiac Histamine Receptors*).

Other tools used to study histamine receptors are receptor agonists and receptor antagonists. These tools, similar to the genetic tools described above, also have limitations, which must be considered in planning experiments. Typical, but not necessarily specific or selective agonists of H_2_R, are listed in Table 1. Obviously, histamine itself is an agonist of all four known histamine receptors. Histamine is therefore also an agonist of H_2_-histamine receptors. It might be of physiological relevance that the affinity of histamine for the four histamine receptors is the lowest for H_2_R. Indeed, histamine has a higher affinity for H_1_-, especially for H_3_- and H_4_-histamine receptors, than for H_2_-histamine receptors (Panula et al., 2015). However, these observations clearly show that histamine is not a specific agonist of H_2_-histamine receptors. If contractile effects of histamine are detected in the mammalian heart, which histamine receptor is involved remains unknown. Specific histamine receptor antagonists must be used to classify the contractile effect of histamine and link it to, for instance, a H_1_-or and H_2_-histamine receptor.

**TABLE 1:** Agonists at H_2_-histamine-receptors.

Agonist name	pD2	Tissue studied	References
Compound 16	9.61	Sf9 insect cells expressing the human H_2_R	Birnkammer et al. (2012)
Apromidine	8.0	Guinea pig isolated right atrial preparations	Buschauer (1989)
BU-E-76	^3^7.91	^1,2^ *In vivo* haemodynamic of guinea pig left ventricle	^1^ Felix et al. (1991a), ^2^ Felix et al. (1995)
		^3^Guinea pig isolated right atrial preparations	
			^3^ Buschauer and Baumann (1991)
BU-E-75	^3^7.90	^1,2^ *In vivo* haemodynamic of guinea pig left ventricle	^1^ Felix et al. (1991a), ^2^ Felix et al. (1995)
		^3^Guinea pig isolated right atrial preparations	
			^3^ Buschauer and Baumann (1991)
Amthamine	7.04	Guinea pig isolated right atrial preparations	Eriks et al. (1992)
Impromidine	7.04	Guinea pig isolated right atrial preparations	Bertaccini and Coruzzi (1981)
4-Methyl-histamine	7.01	pH measurement in isolated perfused rat stomach	Durant et al. (1975)
Dimaprit	^1^6.19	^1^Guinea pig isolated right atrial preparations	^1^ Parsons et al. (1977)
		^2^Guinea pig hippocampal slices	^2^ Garbarg and Schwartz (1988)
		^3^CHO cells expressing the rat H2-histamine-receptor	^3^ Smit et al. (1996a)
Histamine	^1^6.60	Guinea pig isolated right atrial preparations	^1^ Bertaccini and Coruzzi (1981)
	^2^6.00		^2^ Buschauer and Baumann (1991)

Synopsis of some relevant histamine agonists (first column), their affinity at H_2_-histamine receptors (decadic logarithms of their affinity constants, second column), the tissue studied (third column) and the references (fourth column).

The agonists listed in Table 1 are of comparable potency, or, compared with histamine, they are much more potent agonists of H_2_-histamine receptors. The first agonist that was found to act on H_2_R but not on H_1_R was dimaprit (Table 1). Later, in addition to the previously cloned H_1_- and H_2_-histamine receptors, novel H_3_- and H_4_-histamine receptors were cloned. It was found that dimaprit, indeed, did not stimulate cloned H_1_R but stimulated cloned H_2_R. However, dimaprit was shown to stimulate H_3_-and H_4_-histamine receptors even more potently than H_2_-histamine receptors (Panula et al., 2015). Currently, a molecule called compound 16 is known to be one of the most potent agonists of H_2_-histamine receptors (Table 1). Interestingly, in Langendorff-perfused guinea pig hearts, a derivative of dimaprit, called apromidine, exerted a positive inotropic effect, which occurred without changing the heart rate (Felix et al., 1991a, 1995). Two dually fluorinated apromidine derivatives, which are known H_2_R agonists (BU-E-75 and BU-E-76), not only induced a positive inotropic effect but also reduced heart rate in Langendorff-perfused guinea pig hearts or living anaesthetised guinea pigs (Felix et al., 1991a, 1995). The lack of a positive chronotropic effect is puzzling: in isolated spontaneously beating guinea pig right atria, BU-E-75 and BU-E-76 exerted potent positive chronotropic effects: pD_2_-values of 8.12 and 8.05 were compared with pD_2_-values for a positive inotropic effect in isolated paced guinea pig papillary muscles at 7.90 and 7.91, respectively (Buschauer and Baumann 1991). These results clearly showed that BU-E-75 and BU-E-76 are potent agonists of H_2_-histamine receptors in the guinea pig sinus node (SA). However, in another study, the same group reported that the efficacy of inducing a positive chronotropic effect, that is, an absolute increase in the number of heartbeats in Langendorff-perfused guinea pig heart, was less than that induced by impromidine, another dimaprit derivative (Felix et al., 1991a). Similarly, BU-E-75 and BU-E-76 were more effective in inducing a positive inotropic effect on guinea pig ventricle compared with impromidine (Felix et al., 1991a). These authors speculated that *in vivo*, in anaesthetised guinea pig and Langendorff-perfused guinea pig heart, additional effects of BU-E-75 and BU-E-76, such as vagal stimulation, must exist, which explained their negative chronotropic effects (NCE) (Felix et al., 1991a, 1995). They also reported that BU-E-75 and BU-E-76 were virtually non-arrhythmogenic (Felix et al., 1991a).

A caveat is in order at this stage. Even if one uses a specific H_2_R agonist that does not have any measurable affinity for the other three histamine receptors, control experiments are necessary to prove that the histamine receptor agonist does not act on other sarcolemmal receptors that alter cardiac contractility. A sound precaution could be to test a new H_2_R agonist to determine whether one of the well-characterised H_2_R antagonists, such as cimetidine or famotidine (Table 2), abrogates its cardiovascular effects. In Table 2, we have deliberately listed only one H_2_R antagonist, burimamide, which is now only of historical value. Burimamide was the first H_2_R antagonist to be described (Black et al., 1972). Because of its short half-life and poor oral bioavailability, it has never been clinically applied. However, it has been used in many seminal studies to identify cardiac H_2_-histamine receptors. The other H_2_R antagonists shown in Table 2 are still used clinically, and they have been used as substitutes for burimamide to study the functional role of H_2_-histamine receptors in the heart. In Table 2, we present mainly data on affinity derived from cell culture studies in which the authors used human H_2_-histamine receptors to measure affinity. Such data are difficult to obtain in studies on isolated human organs, but, under identical conditions, they should allow for comparisons between several frequently used H_2_R antagonists in cardiovascular research. Figure 1A shows the generally known H_2_R-initiated pathways and the current putative signal transduction steps in the mammalian heart.

**TABLE 2:** Antagonists at H_2_-histamine receptors.

Antagonist name	−lg IC_50_	Inverse agonism	Tissue studied	References
GASTROINTESTINAL DRUGS:				
Cimetidine	6.18	+	Transfected Chinese hamster ovary cells	Baker (2008)
Ranitidine	6.79	+	Transfected Chinese hamster ovary cells	Baker (2008)
Nizatidine	7.10	+	Transfected Chinese hamster ovary cells	Baker (2008)
Burimamide	^1^7.16	−	^1^Transfected Chinese hamster ovary cells ^2^Guinea pig right atrium	^1^ Smit et al. (1996b)
	^2^7.8			^2^ Black et al. (1972)
Zolatidine	7.39	+	Transfected Chinese hamster ovary cells	Baker (2008)
Tiotidine	7.93	+	Transfected Chinese hamster ovary cells	Baker (2008)
Famotidine	8.34	+	Transfected Chinese hamster ovary cells	Baker (2008)
ICI 162846	8.43	+	Transfected Chinese hamster ovary cells	Baker (2008)
PSYCHIATRIC DRUGS:				
Amitriptyline	5.72 or 6.95	+	^1^Neuronal cells	^1^ Kanba and Richelson (1983)
			^2^Baculovirus system	^2^ Appl et al. (2012)
			^3^Langendorff-heart H_2_-TG mouse	^3^ Neumann et al. (2021b)
Imipramine	5.48 or 6.10	+	^1^Neuronal cells	^1^ Kanba and Richelson (1983)
			^2^Baculovirus system	^2^ Appl et al. (2012)
Chlorpromazine	5.5 or 5.81	+	^1^Neuronal cells	^1^ Kanba and Richelson (1983)
			^2^Baculovirus system	^2^ Appl et al. (2012)
Mianserin	5.55 or 6.35	+	^1^Neuronal cells	^1^ Kanba and Richelson (1983)
			^2^Baculovirus system	^2^ Appl et al. (2012)
Haloperidol	4.54 or 5.94	+	^1^Neuronal cells	^1^ Kanba and Richelson (1983)
			^2^Baculovirus system	^2^ Appl et al. (2012)

Synopsis of some relevant histamine receptor antagonists (first column), their affinity (second column, negative decadic logarithm of their inhibitory action) for H_2_-histamine-receptors, their ability to act as inverse agonists (+, third column), the tissue studied (fourth column) and the references (fifth column). With the exception of burimamide all listed drugs are inverse agonists. The upper half consists of antagonists designed to be specific antagonists at H_2_-histamine-receptors and were initially developed to block these receptors in the gastrointestinal tract. The lower half of Table 2 lists drugs used in psychiatry to treat psychosis or depression. In early studies (see text) these compounds were shown to antagonize the stimulatory effect of histamine on the activity of adenylyl cyclases from the guinea pig brain or guinea pig heart. Baker (2008) used human H_2_-histamine receptors for transfection experiments, thus these data are clinically of special relevance and were therefore chosen to be presented here. Lower affinities are from Kanba and Richelson in cells and higher affinity values are from Appl et al. (2012) where recombinant receptors produced in a baculovirus system were used.

## 2 Interaction of H_2_R With Other G-Protein Coupled Receptors

**FIGURE 2:**
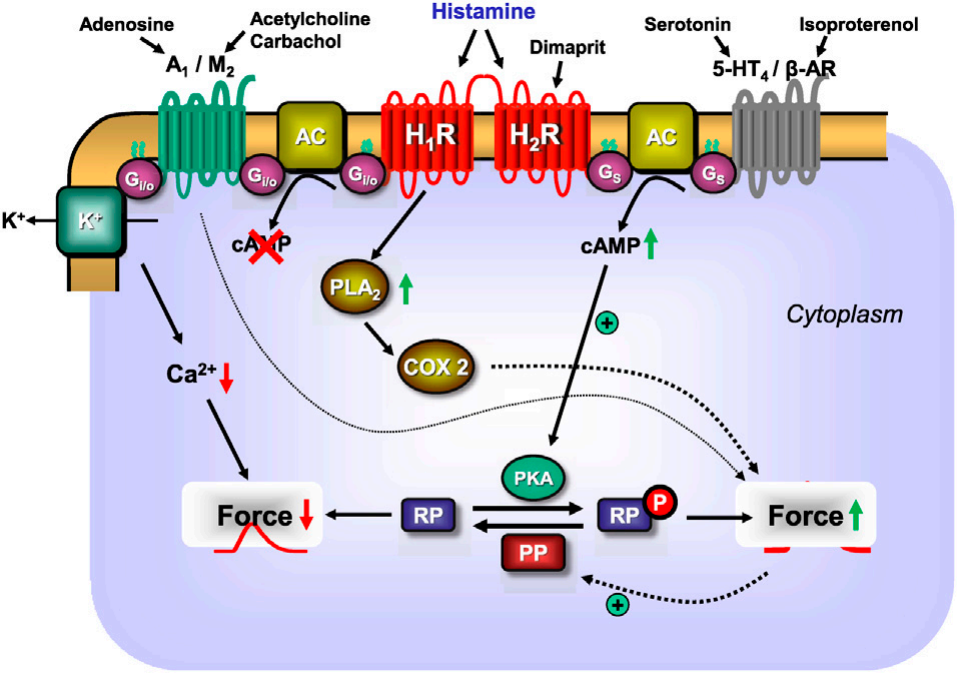
Scheme: putative mechanisms of interaction between H_1_- or H_2_-histamine receptors and other GTP-binding protein-coupled heptahelical receptors in the sarcolemma of a cardiomyocyte. As delineated in Figure 1A, H_2_-histamine receptors after stimulation by endogenous histamine or by the exogenous H_2_-histamine receptor selective agonists like dimaprit will elevate via stimulatory GTP-binding proteins (G_s_) the activity of sarcolemmal adenylyl cyclases (AC). Thus, more cAMP is formed and cAMP-dependent protein kinases (PKA) are activated. This leads to a subsequent phosphorylation and activation of cardiac regulatory proteins (RP). Their phosphorylation (compare Figure 1A for details) leads to an increase in force of contraction. The same pathway is used by the cardiac 5-HT_4_-serotonin receptor stimulated by endogenous serotonin or the β-adrenoceptors (β-AR) stimulated by exogenous isoproterenol to increase cAMP and thereafter force of contraction. The increase of force of contraction induced by histamine by acting on H_2_-histamine receptors is abrogated by additionally acting endogenous compounds like adenosine acting on A_1_-adenosine receptors or endogenous acetylcholine (or exogenous carbachol) stimulating M_2_-muscarinic receptors. Three pathways may be used by M_2_-muscarinic receptors and A_1_-adenosine receptors. They may inhibit via inhibitory G-proteins (G_i/oα_) the activity of AC, thereby reduce cAMP content and thus decrease force of contraction. In addition, A_1_-adenosine and M_2_-muscarinic receptors can activate sarcolemmal potassium ion channels: this shortens the duration of the action potential; less time is available for trigger Ca^2+^ to enter the cell via L-type Ca^2+^ channels (see Figure 1A), cytosolic Ca^2+^ declines and force falls. Lastly, M_2_-muscarinic and A_1_-adenosine receptors may directly or indirectly activate protein phosphatases (PP, see Figure 1A) which would reduce the phosphorylation state and subsequently the force in the myocardium. Moreover, as shown in Figure 1B, H_1_-histamine receptors, may activate phospholipase A2 (PLA_2_), thereby activating cyclooxygenase 2 (COX2) which generates metabolites of arachidonic acid which can elevate force of contraction. Finally, there seems to be a direct interaction whereby the H_2_-receptor stimulation can reduce the activity of the H_1_-histamine receptor.

H_2_R can heterodimerise with H_1_R (Figure 2), which was observed after receptor stimulation in U937 cells (i.e., a macrophage cell line, which per se expresses both receptors) and H_2_R transfected Chinese hamster ovary (CHO) cells, leading to the desensitisation and internalisation of H_2_-histamine receptors in endosomes (Alonso et al., 2013). A functional interaction was produced as follows: in cell culture, H_1_-histamine receptors were stimulated for 60 min. Dimaprit evoked a smaller increase in cAMP (Figure 1A) in these cells than under control conditions. Conversely, dimaprit pre-treatment led to a reduced H_1_R-mediated IP_3_-increase (Figure 1B), indicating functional cross-talk, which was not due to receptor phosphorylation by kinases (Alonso et al., 2013). Whether this kind of heterodimerisation occurs in the heart, particularly in the human heart, and has functional consequences has not yet been studied. However, it could be addressed because both receptors are present on, for instance, guinea pig cardiomyocytes, which was shown in histological results (Matsuda et al., 2004). Many G-protein coupled receptors are known to heterodimerise. Hence, it is conceivable, but unknown, whether H_2_R dimerises with other receptors in addition to H_1_R. Diverse functional, but not necessarily structural, interactions between histamine acting via H_2_-histamine receptors and other cAMP-changing agents have been studied (Table 3). Therefore, the following question arises: What are the results of the interaction of H_2_-histamine receptors with other receptors? One way to address this question, which is also (patho)physiologically relevant, is the following: in isolated Langendorff-perfused heart, histamine was given initially, which increased the force of contraction in the left ventricle. It was also found to increase the current through Ca^2+^ channels in the sarcolemma (Belevych et al., 2004). Adenosine (or carbachol, a stable derivate of acetylcholine and an unspecific agonist at muscarinic receptors) was then applied, which reduced the force of contraction in the heart. This functional inhibition has usually been explained as follows (Figure 2): H_2_R stimulation increases adenylyl cyclase activity (Figure 1A), which is then reduced by the stimulation of A_1_-adenosine receptors or M_2_-muscarinic receptors (Table 3) (Baumann et al., 1981a). The situation is somewhat different in experiments on preparations from the right human or canine atrium, where the positive inotropic effects of histamine and dimaprit, mediated by H_2_R, are also reduced by adenosine or carbachol (Endoh, 1979; Baumann et al., 1981a). However, the mechanism of the action of adenosine is not clear. As the ventricle of guinea pig, adenylyl cyclases might be involved. Thus, alternatively strong lines of evidence (Behnke et al., 1990; Böhm et al., 1986; Böhm et al., 1988a; Gupta et al., 1993; Neumann et al., 1994; Herzig et al., 1995; Neumann et al., 1995) have shown that the effects of A_1_-adenosine receptor stimulation or M_2_-muscarinic receptor stimulation occur via subunits of GTP-binding proteins, which leads to the opening of atrial potassium channels without the involvement of cAMP. Thus, a reduction in the action potential (AP) duration and a subsequent negative inotropic effect (NIE) will ensue (Figure 2; Table 3). Because adenosine is released in ischaemia, this functional interaction might be regarded as an antihistaminergic effect of adenosine (Genovese et al., 1988). The opposite interaction was noted, at least under certain conditions: the current in sarcolemma or the force of contraction, both of which were stimulated by β-adrenoceptor agonists, was reduced by the additional application of histamine (Belevych et al., 2004; Gross et al., 1984). These results indicate that H_2_-histamine receptors couple in the heart not only via stimulatory GTP-binding proteins to activate adenylyl cyclase but also via inhibitory GTP-binding proteins to inhibit the activity of adenylyl cyclase (Figure 2). This behaviour is not without precedence, and it is well known in the β_2_-adrenoceptor. Similarly, the mode of interaction between H_2_-histamine receptors and other receptors, such as purinoceptors, may depend upon the signal pathway involved. An adenosine triphosphate (ATP)-induced increase in arachidonic acid in H_2_R transfected cells was inhibited by the additional application of histamine, but the ATP-induced increase in Ca^2+^ was not affected by the application of histamine (Traiffort et al., 1992). This ATP-based interaction has not been studied in the heart. Further evidence indicates that the order of drug application is important for H_2_-histamine receptors and other receptors that are coupled to the activity of adenylyl cyclase. For example, if the cardiac serotonin 4 (5-HT_4_) receptor (i.e., the receptor mediating the positive inotropic effect of serotonin in the human heart) was first stimulated, then H_2_R activation decreased the force of contraction but not vice versa (Neumann et al., 2019; Neumann et al., 2021d). These data are in line with the assumption that H_2_-histamine receptors are coupled via inhibitory and stimulatory G-proteins with the activity of adenylyl cyclase in the heart.

**TABLE 3:** Alterations of histamine-induced effects in the heart.

Histamine-stimulated effect	Functional antagonist	System	Alternative agonist	References
positive chronotropic effect	inhibited by adenosine	human right atrium	or dimaprit	Genovese et al. (1988)
positive chronotropic effect	inhibited by carbachol	human right atrium	or dimaprit	Genovese et al. (1988)
positive inotropic effect	inhibited by adenosine	human right atrium	or dimaprit	Genovese et al. (1988)
positive inotropic effect	inhibited by carbachol	human right atrium	or dimaprit	Genovese et al. (1988)
positive inotropic effect	inhibited by adenosine	human left papillary muscle	or dimaprit	Genovese et al. (1988)
positive inotropic effect	inhibited by carbachol	human left papillary muscle	or dimaprit	Genovese et al. (1988)
positive inotropic effect	inhibited by adenosine	Guinea pig: Langendorff		Baumann et al. (1981a)
positive inotropic effect	inhibited by carbachol	Guinea pig: Langendorff		Baumann et al. (1981a), Baumann et al. (1981b)
adenylyl cyclase	inhibited by adenosine	Guinea pig: Langendorff, canine ventricle		Baumann et al. (1981a), Baumann et al. (1981b), Endoh (1979)
L-type Ca^2+^-channels	inhibited by iso-prenaline	Guinea pig cardiomyocytes		Belevych et al. (2004)
L-type Ca^2+^-channels	inhibited by adenosine	Guinea pig cardiomyocytes		Belevych et al. (2004)
L-type Ca^2+^-channels	inhibited by acetylcholine	Guinea pig cardiomyocytes		Levi and Alloatti (1988) Belevych et al. (2001)

Here, effects probably mediated by H_2_-histamine receptor stimulation are listed (first column) with special regard to their additive or inhibitory interaction with other receptor-mediated effects that are present in the heart. The effects are listed in the first column, the interacting agent in the second column, the tissue and species reported upon in the third column. In the fourth column it is mentioned whether dimaprit in the reference. This was done because in contrast to histamine, dimaprit does not act on H_1_-histamine receptors and thus dimaprit-induced effects are probably H_2_-histamine receptor-mediated.

## 3 Regional Expression of Histamine Receptors in the Heart

**FIGURE 3:**
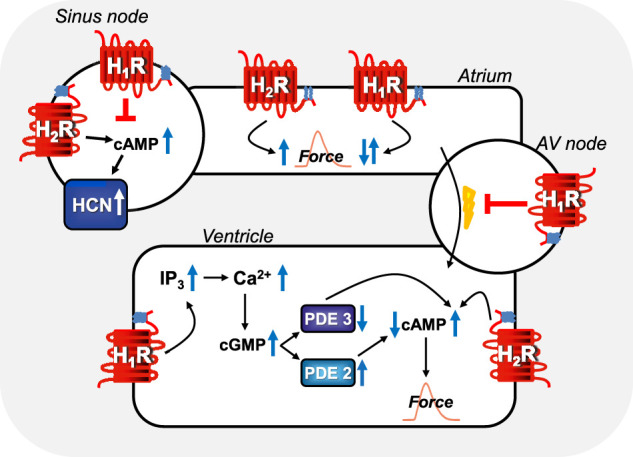
Comparison of regional H_1_-histamine receptor and H_2_-histamine receptor signaling in various regions of mammalian hearts. In sinus node cells, H_2_-histamine receptors can stimulate cAMP-production, this cAMP binds to HCN (=I_f_-currents, hyperpolarization-activated ion channels) which thereafter open more often and tachycardia ensues (see Table 7 for details). Alternatively, H_1_-histamine receptors, in sinus node cells can reduce the beating rate via still unknown mechanisms (see Table 7 for details). In atrial muscle cells, H_2_-histamine receptors (via cAMP, see Figure 1A) and H_1_-histamine receptors (see Figure 1B via, for instance, activation of PLC and thereafter formation of IP3 and/or diacylglycerol and subsequent phosphorylation steps) can both increase atrial force of contraction in some species. In other species, H_1_-histamine receptors in atrial muscle cells decrease force of contraction by activation of phosphodiesterase, inhibition of protein kinases and/or activation of phosphatases. In the atrioventricular (AV) node, H_1_-histamine receptors in most species inhibit electrical conduction into the ventricle (see Table 7 for details). Likewise, in the ventricular muscle cells, H_2_-histamine receptors increase force of contraction by the mechanism depicted in Figure 1A. But also, in some species, ventricular muscle H_1_-histamine receptors can increase force of contraction (see Figure 1B), in other species, H_1_-histamine receptors lead to a reduction in force of contraction via the hypothetical mechanism depicted: a cGMP-mediated increase in phosphodiesterase II (PDE II) activity. Alternatively, in other species cGMP might inhibit PDE III and thereby increase cAMP and subsequently (See Figure 1B for details) force of contraction.

Figure 3 and Table 4 show overviews of the functional actions of histamine in the hearts of several species and in different cardiac regions (Figure 3). Table 4 shows regional differences in the presence and role of H_2_-histamine receptors, which must be considered in planning studies. For comparison, animals that are seldom used in experimental medicine were included in Table 4; for example, H_2_-histamine receptors are used in the python heart. Perhaps it could be concluded that histamine receptors occurred late in the evolution of the animal kingdom. Some aspects of human H_2_R pharmacology are better studied in guinea pigs, and others are better studied in pigs and dogs (Table 4).

**TABLE 4:** Cardiac effects of histamine.

Species	Left atrium	Right atrium	Atrioventricular node	Ventricle	References
Man	PIE: ^3^H_2_	PIE: ^1,2,3,5,8^H_2_ PIE: ^5^H_1_ NIE: ^1,6^H_1_ PCE: ^1,4,7^H_2_ NCE: ^1^H_1_ cAMP: ^5^H_1,2_ PKA: ^5^H_1,2_ cGMP: ^5^H_1_^4^Arrhythmias	^7^AV-block: H_1_	PIE: ^3,7^H_2_, ^5^H_1_ NIE: ^6^H_1_	^1^ Genovese et al. (1988)
					^2^ Zerkowski et al. (1993) (both)
					^3^ Ginsburg et al. (1980)
					^4^ Levi et al. (1981)
					^5^ Sanders et al. (1996) (both)
					^6^ Guo et al. (1984)
					^7^ Vigorito et al. (1983) (both)
					^8^ Graver et al. (1986)
Cat	^9^PIE: NE-release	^9^PCE: H_2_, β: release of NE	n. d	^9^PIE: NE-release	^9^ Laher and McNeill (1980c) (both)
Rabbit	PIE: ^10,11^H_2_ H_2_: ^10,11^cAMP H_1_: ^11^IP_3_^10^H_1_: cGMP H_1_: ^11^no effect on force	PCE: ^10^H_2_^10^H_2_: cAMP ^10^H_1_: cGMP	AV-block H_1_	PIE: ^10^H_2_ (weak) PIE: ^10^H_1_ (strong) ^10^H_1_: cGMP ^12^H_1_: IP_3_^10^PIE: H_1_ > H_2_	^10^ Hattori et al. (1988a)
					^11^ Hattori et al. (1991a)
					^12^ Hattori et al. (1994) (both)
Dog	no effect PIE: H_1_	PCE: ^13,16^H_1_ PIE: ^13^H_1_ release of NE via H_3_ or H_4_? or H_1_	^16,19^AV-block H_1_	^13^No effect *in vivo* NIE: H_1_^15^PIE ^15^cAMP	^13^ Vidrio and Priola (1990) (both)
					^14^ Chiba (1976)
					^15^ Endoh (1979) (both)
					^16^ Flacke et al. (1967) (both)
					^17^ Powell and Brody (1976)
					^18^ Li et al. (1998)
					^19^ Hashimoto (1925)
Guinea pig	PIE: ^29,32^H_1_: ^23^IP_3_ PIE: ^20,28,30^H_1_ NIE: H_1_ and H_2_ Neonatal: PIE H_2_^29^PIE: via NE	PCE: ^21,22,27^H_2_ cAMP: ^21^H_2_ PIE: ^21^H_2_^29^PCE via NE	^27^AV-block: H_1_	PIE:^21,22,27, 32,12^ H_1_ Neonatal: only H_2_ mediated PIE cAMP: ^21,32^ H_2_ PIE: ^21,32,12^H_2_^12^IP_3_: H_1_^25^negative inotropic effect: H_1_	^20^ Hattori et al. (1994) (both)
					^21^ Verma and McNeill (1977) (both)
					^22^ Macleod et al. (1986) (both)
					^23^ Sakuma et al. (1988) (male)
					^24^ Kiniwa and Tasaka (1989)
					^25^ Zavecz and Levi (1978) (male)
					^26^ Hattori et al. (1991b) (both)
					^27^ Levi and Kuye (1974) (male)
					^28^ Hattori and Kanno (1985) (both)
					^29^ Laher and McNeill (1980b) (both)
					^30^ Hattori et al. (1988b) (both)
					^31^ Houki (1973)
					^32^ Shigenobu et al. (1980) (male)
Rat	^36^PIE: NE-release ^3,35^NIE	^36^PIE: NE-release ^36^NCE: Acetylcholine-release	?	^35^NIE: PIE: NE-Release ^33,34^No effect	^33^ Dai (1976) (male)
					^34^ Wellner-Kienitz et al. (2003) (both)
					^35^ Bartlet (1963)
					^36^ Laher and McNeill (1980a) (both)
					^37^ Went et al. (1952)
Mouse	^40^PIE ^41^NIE: H_2_^42^H_2_: cAMP ^38,39,41^no effect	^42^PCE: H_2_	n.d	^38,39^no effect	^38^ Gergs et al. (2019)
					^38^ Gergs et al. (2020) (both)
					^40^ Liu et al. (2002)
					^41^ Goren et al. (1993)
					^42^ Goren et al. (1994)
					^43^ Goren et al. (1995)
Pig	PIE: ^44^H_2_ NIE: ^44^H_1_			*In vivo*: 45PIE:H_2_ *In vivo*: 45NIE: H_1_ PIE: ^44^H_1_ NIE: ^44^H_1_	^44^ Du et al. (1993) (both)
					^45^ Cooper et al. (1995) (both)
Ferret				PIE: Papillary muscle: _i_Ca^2+^, cAMP	^46^ Hurrell et al. (1993) (male)
Chicken		PCE: H_2_		No effect ?	^47^ Kiniwa and Tasaka (1989)
Four-striated snake		PCE: H_2_		PIE: H_2_	Kiniwa and Tasaka (1989)
Soft-shelled turtle		PCE: H_1_		PIE:H_1_	Kiniwa and Tasaka (1989)
Pond turtle		no effect		no effect	Kiniwa and Tasaka (1989)
Fish e.g. common carp		no effect		no effect	Kiniwa and Tasaka (1989) but see also for exceptions ^48^ Reite (1972)
Bullfrog		no chronotropic effect		PIE: H_2_	^49^ Einis (1913)
Crocodile		PCE: H_2_		PIE: H_2_	Kiniwa and Tasaka (1989)
Python	*In vitro* and *in vivo*	PCE: H_2_		PIE: H_2_	^50^ Skovgaard et al. (2009)

In this table, H_1_- or H_2_-histamine receptor-mediated contractile effects in several regions (first row) of relevant (for clinically oriented research) mammalian species (first columns) have been compared. It is apparent that for some species and regions H_2_-histamine receptor are unimportant, partially important or solely important for the cardiac contractile effects of histamine. This has also to be taken into consideration when planning studies or translating them to humans. PIE: positive inotropic effect to histamine or its derivatives; PCE: positive chronotropic effect to histamine or its derivatives; NIE: negative inotropic effect to histamine or its derivatives; a question mark indicates that some uncertainty concerning the nature of the histamine receptor involved exists. NE-release indicates that histamine induces the release of noradrenaline from probably sympathetic varicosities in the cardiac preparations and then NE, activates β-adrenoceptors (β) thus indirectly increasing contractility. The second messengers probably involved in the signal transduction of the histamine receptors (see also Figure 2) are given as cAMP, cGMP, or IP_3_. H_1_ and H_2_ stand for H_1_-histamine receptors and H_2_-histamine receptors and indicate that we think these receptors mediate the change in force or beating rate or increase in the level of the second messenger which follow the receptor name. AV-block means atrioventricular block of conduction in the heart. H_2_-TG, indicates transgenic mice with heart-specific overexpression of the H_2_-histamine receptor. H_1_ > H_2_ is meaning that H_1_-histamin receptor function dominates over H_2_-histamine receptor function. AV-block: H_1_; indicates that histamine induces an atrioventricular block which is H1-histamine receptor mediated. H_2_ cAMP, or H_1_ cGMP, or H_1_ IP_3_ reads that stimulation of the H_2_-histamine receptor or of the H_1_-histamine receptor in this species and cardiac region is known to raise the level of cAMP, or cGMP, or IP_3_, respectively in this tissue. Unless state otherwise, these data refer to isolated cardiac preparations. In canine studies, Chiba injected histamine, 0.3–100 μg, into the cannulated sinus node artery of the isolated right atrium which was blood perfused by a living donor dog (the sex of the dogs was not published: Chiba, 1976). In living anaesthetized dogs on bypass, histamine 0.1–100 mg was intracoronarilly applied (Vidrio and Priola, 1990). In dog lung preparations with blood obtained from donor hearts, histamine (calculated as free base) was intravenously given at doses of 0.1–10 mg (Flacke et al.,^1967^).

In living pigs, histamine hydrochloride solution was infused intravenously at a rate ranging from 0.5 to 10 µg per kilogram body weight per minute and they measured left ventricular pressure via an intraventricular catheter (Cooper et al., 1995). At low concentration of histamine they noted a negative inotropic effect and at high concentration they measured a positive inotropic effect that was antagonized by ranitidine. In patients, histamine hydrochloride was pumped at a rate of 0.4 µg per kilogram body weight per minute into the left antecubital vein (Vigorito et al., 1983).

The symbol “β “indicates that for instance the positive chronotropic effect of histamine in cat heart is in part blocked by application of a β-adrenoceptor antagonist, suggesting the mediation of that effect via β-adrenoceptors.

In the column with references in brackets available information on sex of animals or human patients were given: male: male animals; both: both genders were used. In some publications, even on humans, sex was not published and therefore is not listed here.

**FIGURE 4:**
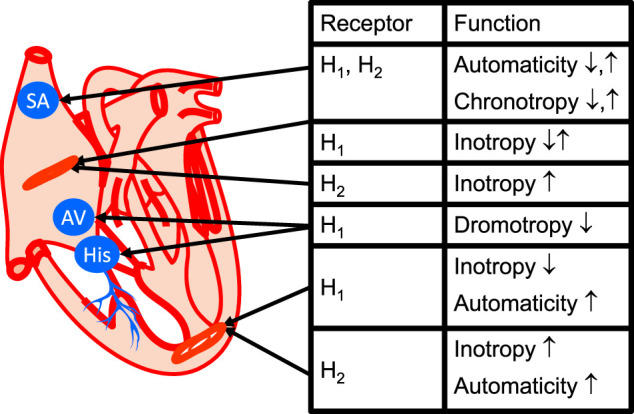
Cardiac conducting system and regional histamine receptor expression in the heart (modified from Stein et al., 1998). Here, one has tried to relate the mechanical information in Figure 2 with anatomically correct location of the receptor. In the sinus node (SA), the H_2_-histamine receptor when it is expressed probably also increase chronotropy, that is increases the heart rate. If the H_1_-histamine receptor is functional, if can decrease but sometimes also increase the heart rate: this is meant by ↓ and ↑ (see Table 4 for species differences). For simplicity, in the ventricle a negative inotropic effect of H_1_-histamine receptor activation is only depicted. However, in some species a positive inotropic effect of H_1_-histamine receptor activation has been described (compare Table 4). If a functional H_2_-histamine receptor is expressed in the atrium or ventricle it always increases inotropy (Table 4). Also indicated is the proarrhythmic effect of H_2_-histamine receptor stimulation in the ventricle by indicating increased automaticity. H_1_-histamine receptors, if present in the AV node (AV), always seem to have negative dromotropic effects, that is, they slow the conduction through the AV node (Table 7). Here, also His-bundles (HIS) are shown where a decrease in the conduction time via H_1_-histamine receptors can sometimes be measured.

Several types of cardiomyocytes conduct the heart beat in the different regions in the heart. Sinus node cells act as a cardiac pacemaker, and atrial cardiomyocytes form the main bulk of atrial muscle. Specialised ventricular cardiomyocytes form the path of the conducting system, which propagates depolarisation starting at the sinus node via specialised cells in the atrium (Bachmann bundles) via the atrioventricular node cells, the His-bundle, the Tawara branches, and the Purkinje fibres in the ventricle walls (Figures 3, 4). However, few histological studies have been conducted to examine histamine receptors. A seminal study that used semiquantitative immunohistochemistry revealed a high density of H_1_-histamine receptors on sinoatrial nodal cells and cells in the atrioventricular node but less expression in the surrounding atrial or ventricular myocardium of guinea pig (Matsuda et al., 2004). These authors detected H_2_-histamine receptors immunologically mainly in the working myocardium of the right atrium and the ventricular cells in proximity to the atrioventricular cells in guinea pigs (Matsuda et al., 2004). There are no published comparative studies on the histology of the human heart; therefore, this topic warrants future research. Alterations of H_2_-histamine receptors in cells in this pathway are expected to be of huge clinical relevance, as they can certainly lead to various cardiac arrhythmias. Alterations of H_2_R expression might be relevant for not only primary arrhythmias because of inborn errors but also secondary arrhythmias upon ischaemia, hypertrophy, drug treatment, and perhaps ageing. However, further research in this regard needs to be undertaken.

Concerning the expression (Figures 1, 3, 4 and Table 5) and the cellular heterogeneity of H_2_-histamine receptors in the heart, H_2_-histamine receptors have long been known to be present and functional in blood cells. These blood cells are pumped into the heart and continuously removed by circulation. Specifically, H_2_R is expressed on leucocytes, macrophages, mast cells (Marquardt et al., 1994), neutrophils (Fredholm et al., 1999), thrombocytes, and erythrocytes (Table 5). In histological studies with antibodies, the specificity of which is poor and a research need (Seifert et al., 2013) or messenger ribonucleic acid (mRNA) detection), H_2_-histamine receptors have been identified in blood containing cardiac tissue section samples or cardiac homogenates. However, measurements of H_2_-histamine receptors in cardiac homogenates reveal their expression in all cell types present in the heart. It could be assumed that bands thought to be specific to H_2_R in Western blots, which are made from whole heart homogenates, mainly arise from cardiomyocytes. This assumption, however, does not necessarily hold true unless it is repeated with homogenates from purified cardiomyocytes (Gergs et al., 2019; and own unpublished observations). Hence, some data in the literature on cellular expression of H_2_R proteins await confirmation.

**TABLE 5:** Localization of H_2_-histamine receptors.

Tissue	Species	Tissue/Cell type: References
1. Cardiomyocytes		
1.1	Adult rat	Whole heart: Matsuda et al. (2004) Zeng et al. (2014)
	Neonatal rat	Cardiomyocyte: Zeng et al. (2014)
1.2	Pig	Ventricle: Cooper et al. (1995)
1.3	Human	Atrium and ventricle: Matsuda et al. (2004)
1.4	Mouse	Ventricle: Lacking: Gergs et al. (2019): Present: Fitzsimons et al. (2001)
1.5	Rabbit	Ventricle: Hattori et al. (1991a), (1991b)
1.6	Guinea pig	Ventricle: Agata et al. (2010), Matsuda et al. (2004)
2. Blood cells		
2.1	Human	Platelets: Nakamura et al. (1999)
2.2	Human	Mast cells: Bachert (2002)
2.3	Human	Macrophages: Jutel et al. (2009)
2.4	Human	Neutrophils: Busse and Sosman (1976)
2.5	Human	Erythrocytes: Wagner et al. (2006)
3	Human	Vascular smooth muscle cells: Ottoson et al. (1988)
4	Human	Endothelial cells: Luo et al. (2013)
5	Human	Lymphocytes: Jutel et al. (2009)
6	Human	Basophils: Bachert (2002)
7	Rat	Fibroblasts: Zeng et al. (2014)

Here, the tissue distribution and localization of H_2_-histamine receptors in different cell types (first column) present in the heart of several species (second column) or blood constituents (third column) are listed. It is apparent that H_2_-histamine receptors are by no way confined to mast cells but are present on several cell types. SMC: smooth muscle cells. EC: endothelial cells. It is worth mentioning that whereas the H_2_-histamine receptor is found biochemically in the adult rat heart, it is only functional in neonatal and possibly fetal rat heart when one compares this table with Table 4. In the mouse, H_2_-histamine receptors were present in wild-type cardiomyocytes as messenger ribonucleic acid by polymerase chain reaction but were functionally absent even in electrically stimulated adult cardiomyocytes (Gergs et al.,^2019^).

## 4 Expression, Interaction, and Desensitisation of H_2_R

### 4.1 Brief Notes on H_2_R Biochemistry

The homology of mouse and human H_2_-histamine receptors at the protein level is about 85% (Kobayashi et al., 1996). The three-dimensional structure of the H_2_R has been studied using virtual crystallisation (Conrad et al., 2020; Hok et al., 2020). Histamine has been observed to bind to amino acids in transmembrane domains three and five or six (Panula et al., 2015). However, to the best of our knowledge, crystallisation data on human H_2_-histamine receptors alone and binding to a H_2_R agonist or binding to a H_2_R antagonist are currently not available (Hok et al., 2020).

Several transcription initiation sites of the promoter of the human H_2_R gene and variable 3′-untranslated regions have been characterised (Murakami et al., 1999). These transcript variants are thought to explain, at least in part, the up-and-down regulation of receptors and their differential expression. Only a few data on the altered expression of H_2_R in the human heart are available. However, in the heart of a special transgenic mouse, the expression of H_2_R at the mRNA level and protein level was decreased (Fitzsimons et al., 2001). In this mouse model, histidine decarboxylase, which is the main enzyme responsible for the production of histamine, was deleted in all tissues (Fitzsimons et al., 2001). These data are proof of the principle that the transcriptional regulation of H_2_R can occur in mammalian hearts. However, this field is largely unexplored and requires further research.

It is well known that even a single amino acid mutation can alter the ligand affinity of G-protein coupled receptors. The same principle applies to H_2_-histamine receptors. Indeed, mutations to dissect the ligand binding sites and the sequences involved in signal transduction of the H_2_R have been widely studied (Panula et al., 2015). For instance, the expression of a C-terminally truncated variant of H_2_R was found to lead to more generation of cAMP compared with the expression of wild-type (non-mutated) H_2_-histamine receptors (Fukushima et al., 1997) in transfected cells in culture, which may therefore be regarded as a gain in function mutation. Further studies on mutations revealed that G-protein coupled receptor kinase 2 and 3 (GRK2 and GRK3) in COS-7 cells (a fibroblast-like cell line) led to the desensitisation of H_2_R in histamine (Rodriguez-Pena et al., 2000). It would be interesting to overexpress these mutated H_2_-histamine receptors in the mouse heart and determine whether a gain in function or the histamine-induced desensitisation of force of contraction in the heart were regulated in a fashion similar to transfected non-muscle cells. As previously discussed in this paper, the isolated heart of wild-type mice does not react to histamine: wild-type mice have no functional histamine receptors that increase beating rate or force of contraction. It could be argued that a mutated H_2_R in mouse heart could be practically overexpressed on a “knock out” baseline; hence, it should be possible to study mutations in comparison with the hearts of wild-type mice (Gergs et al., 2019). At present, it is unknown why the mouse heart does not display inotropic or chronotropic effects of exogenously applied histamine. Indeed, the mRNA and protein of H_2_R are present in mouse heart (Fitzsimons et al., 2001; Gergs et al., 2019). However, the lack of effect of H_2_R on mouse heart is not an isolated curiosity. Similarly, the mRNA and protein of H_2_R were present in the hearts of rats (Matsuda et al., 2004).

### 4.2 Interactions Between Histamine, Histamine Receptors, and Noradrenaline

Any positive inotropic effects of histamine in rat cardiac preparations vanished when the animals were pre-treated with reserpine or studied in an organ bath in the continuous presence of β-adrenoceptor antagonists such as propranolol (Laher and McNeill 1980a). These experimental findings are consistent with the explanation that in rats, histamine receptors release noradrenaline, which stimulates β-adrenoceptors that increase the force of contraction (Laher and McNeill 1980c). These actions of noradrenaline are impossible if the animals are pre-treated with reserpine because it is known to lower the noradrenaline content in the heart and if the tissue contains β-adrenoceptor antagonists such as propranolol (Laher and McNeill 1980c). We have noted that a single bolus of 100 µM of histamine, non-cumulatively applied in isolated electrically stimulated left atrial preparations of wild-type mice in an organ bath exerted a small but reproducible positive inotropic effect, which is absent in the presence of propranolol or after pre-treatment of mice with reserpine (Gergs et al., 2019 and unpublished observations). Hence, in rat and mouse hearts, H_2_-histamine receptors are either not present on cardiomyocytes, or they do not couple with pathways that increase the force of contraction or heartbeat.

Interestingly, at least in guinea pig left atrial preparations, a biphasic effect of histamine was observed. When histamine was not cumulatively but sequentially applied, a fast initial increase in force was followed by a slower increase in the force of contraction (Wilson and Broadley 1980). 2-Methyl-histamine, another typical H_1_R agonist (Black et al., 1972), and 2-pyridylethylamine (PEA) in the presence of propranolol (to rule out indirect effects of histamine on β-adrenoceptors via noradrenaline release) also elicited a biphasic positive inotropic effect in isolated left atrial preparations in guinea pigs. These biphasic effects were more prominent at 25°C than at 37°C in an organ bath (Wilson and Broadley 1981a). At 25°C in the organ bath, the first peak in force generation was dissolved in the presence of the H_1_R antagonist mepyramine, but the second peak was maintained (Wilson and Broadley 1981a), which prompted the authors to predict that a possible novel histamine receptor was involved, which, however, was apparently never fully clarified (Wilson and Broadley 1981b). In isolated right atrial guinea pig preparations, blocking H_2_-histamine receptors by cimetidine revoked the positive inotropic effect of histamine, but the histamine exerted a biphasic effect on the force of contraction. The biphasic pattern was explained by an intermediate negative inotropic effect of histamine mediated via H_1_-histamine receptors (Wilson and Broadley 1981b) (Table 4; Figure 1B). These findings might be regarded as evidence that even in the same region of the heart, histamine uses different histamine receptors.

### 4.3 Homologous and Heterologous Desensitisation and Sensitisation of the H_2_R

The desensitisation of H_2_R in the native cells of various species and in transfected cells using human, monkey, rat, or canine H_2_-histamine receptors expressed in transfected non-muscle cells has repeatedly and consistently been reported. These studies used the cellular cAMP content to determine the cellular response to histamine and to identify the receptor involved by using specific agonists and antagonists (Schreurs et al., 1984; Arima et al., 1993; Smit et al., 1994, 1996a, 1996b; Lemos Legnazzi et al., 2000; Fernandez et al., 2008; Fukushima et al., 1993). In one step in studying the desensitisation of human H_2_R in the human heart, functional homologous desensitisation in human H_2_R-expressing mice heart has been recently reported (Gergs et al., 2019). Interestingly, cross desensitisation was also observed to occur: in cell culture, the stimulation of H_1_-histamine receptors attenuated the H_2_R agonist-mediated increase in cAMP levels (Fernandez et al., 2011; Alonso et al., 2013). Translating these findings to clinical application could predict that desensitisation is expected in patients undergoing long-term therapy with H_2_R agonists or suffering tumours (e.g., phaeochromocytoma) in which histamine is produced. In a clinical setting, histamine is given parenterally to treat certain types of haematological tumours (Grauers Wiktorin et al., 2019), but, to the best of our knowledge, studies on cardiac desensitisation in these patients have not yet been published. Using cAMP as read out, sensitisation or even resensitisation after desensitisation by the application of H_2_R antagonists such as cimetidine (Table 2) or ranitidine (Table 2) in CHO cells or by removing an H_2_R agonist have been reported (Smit et al., 1996b; Alewijnse et al., 1998). Intriguingly, the heterologous sensitisation of human cardiac H_2_-histamine receptors has been measured in human atrial cardiac strips in patients treated with β-adrenoceptor blockers for some time prior to cardiac surgery and compared with patients without β-adrenoceptor blockage (Sanders et al., 1996). The authors observed that in isolated electrically stimulated human right atrial muscle strips, there was an enhanced (increased potency and efficacy) contractile response to histamine (Sanders et al., 1996). However, the clinical relevance of their findings is still under speculation, and they might warrant further research effort because the density of H_2_-histamine receptors at the mRNA or protein level was not reported (Sanders et al., 1996). Moreover, it would be interesting to know the incidence of arrhythmias in these patients prior to cardiac operation. Two different pharmacological effects would be in play: the proarrhythmic effect of more sensitive H_2_-histamine receptors and the anti-arrhythmic effect of the β-adrenoceptor antagonist, which might cancel each other out in a living patient with an intact vegetative nervous system. Mechanistically, it seems relevant that the overexpression of H_2_-histamine receptors in mouse heart increased the incidence of supraventricular arrhythmias in isolated right atrial preparations in these animals in an organ bath (Neumann et al., 2021b). This finding suggests that the increased density of H_2_-histamine receptors in patients might be caused by supraventricular arrhythmias. Furthermore, it could be speculated that in these patients, H_2_R antagonists may prevent such supraventricular arrhythmias.

Mutations in other regions of the H_2_R revealed that different sequences are involved in receptor desensitisation apart from receptor internalisation (Rodriguez-Pena et al., 2000). Some mutations of human H_2_R have been correlated with various diseases, such as stomach carcinoma, schizophrenia, asthma, allergies, and Morbus Parkinson (Orange et al., 1996; Ito et al., 2000; Jones and Kearns 2011; Arisawa et al., 2012). However, to the best of our knowledge, a significant correlation between mutations of the H_2_R and cardiac disease has not yet been reported. Recently, RNA sequencing in the human heart identified the H_2_R directly on the RNA level, as well as a splice variant that might be relevant for the manifestation of cardiac hypertrophy (Leary et al., 2018a) (see below).

## 5 Signal Transduction of Cardiac Histamine Receptors

**FIGURE 5:**
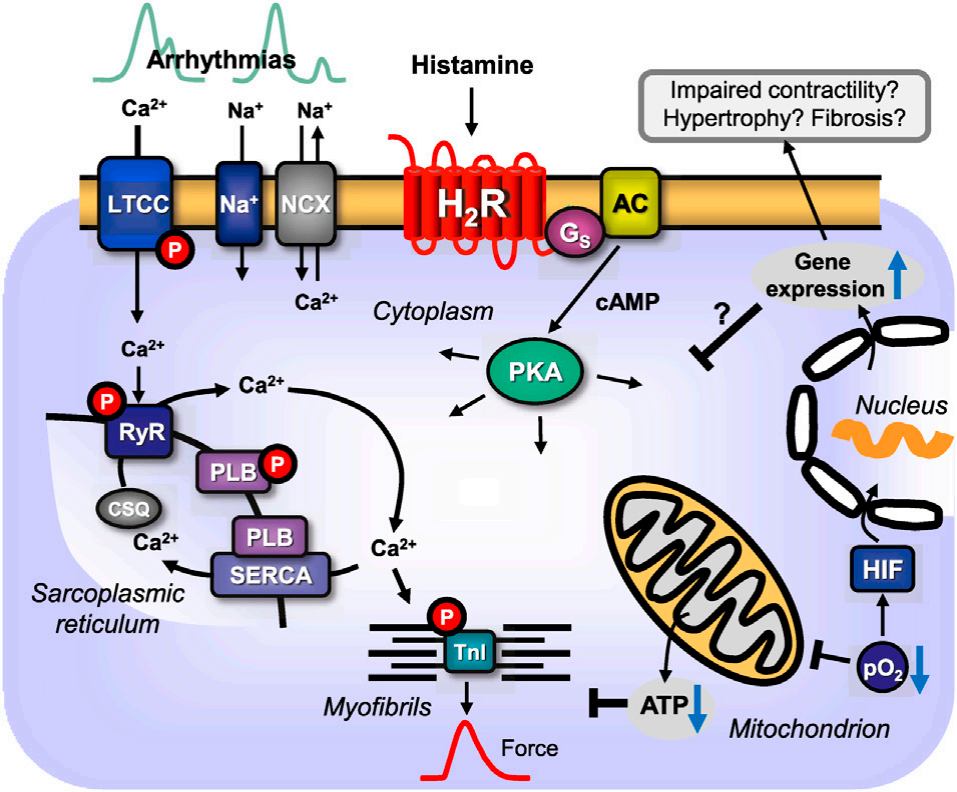
Scheme: putative pathophysiological role(s) of cardiac H_2_-histamine-receptors (H_2_R). H_2_R via stimulatory GTP-binding proteins (Gs) can activate adenylyl cyclases (AC) which would enhance the 3′,5′-cyclic adenosine-phosphate (cAMP)-levels in central compartments of the cardiomyocyte and activate cAMP-dependent protein kinases (PKA), which would increase the phosphorylation state and thereby the activity of various regulatory proteins in the cell (see Figure 1A). PKA-stimulated phosphorylation might also increase the current through the L-type Ca^2+^ channel (LTCC) and/or release of Ca^2+^ from the sarcoplasmic reticulum (SR) via the cardiac ryanodine receptor (RYR), which can occur in a non synchronous way that leads to early **(top left)** or delayed **(top right)** afterdepolarizations and thus to arrhythmias. In diastole, Ca^2+^ is pumped via the SR-Ca^2+^-ATPase (SERCA) from the cytosol into the SR. Activity of SERCA is increased by phosphorylation of phospholamban (PLB). PKA can enhance nuclear gene transcription. In this context, the expression of putatively detrimental proteins may be enhanced and that may impair cardiac function by fostering fibrosis and hypertrophy, reduce cardiac contractility and may lead to heart failure. Hypoxia (reduced oxygen partial pressure: pO_2_) and ischaemia impair respiration in the mitochondrion and thus formation of ATP in mitochondria or might activate directly hypoxia-inducible transcription factors (HIF). Increased expression or altered function of sarcolemmal ion channels like the sodium cation channel (Na^+^) or the sodium/calcium exchanger (NCX) but also increased expression of H_2_-histamine receptors, can lead to supraventricular or ventricular arrhythmias by alteration of Ca^2+^ homeostasis.

The signal transduction (Figure 1A) of H_2_R in general also involves binding to stimulatory G-proteins (Gs-proteins) in peripheral tissues (Table 6). When generated, cAMP then activates a cAMP-dependent protein kinase (PKA), which then phosphorylates typical targets in the heart (Figure 1A). Some of these targets are still hypothetical substrates, such as the ryanodine receptor, whereas others have been shown in transgenic mice (phospholamban, phosphatase inhibitor 1) (Gergs et al., 2019, 2020, 2021b; Neumann et al., 2021d). Moreover, H_2_R stimulation can increase the phosphorylation state of the inhibitory subunit of troponin (TnI) and the myocardial C-protein. Observations in H_2_-TG (transgenic mice with heart-specific overexpression of the H_2_R) have remained unpublished. In the isolated human atrium, H_2_R stimulation increased cAMP content (Sanders et al., 1996), the activity of PKA (Sanders et al., 1996), the phosphorylation state of phospholamban on serine 16 (Neumann et al., 2021a), which is phosphorylated by PKA, and the phosphorylation state of phospholamban on threonine 17 (Neumann et al., 2021a), which is phosphorylated by a Ca^2+^ calmodulin-dependent protein kinase (CaMKII). The phosphorylation of phosphatase inhibitor 1 (Gergs et al., 2019) was observed to activate this protein, which then inhibited protein phosphatase 1, a major cardiac phosphatase (Figure 1A) (Herzig and Neumann 2000), thus amplifying and possibly prolonging the effect of PKA on protein phosphorylation in the heart. Phosphatase 1 showed a highly complicated compartmentalisation in the heart (Herzig and Neumann 2000; Liu, 2021), and thus histamine pathways might be fine-tuned. H_2_-histamine receptors not only increased phosphorylation via PKA but also via β-arrestin (Figure 1A) and other transducers, which finally increased the phosphorylation state and activity of downstream kinases, such as extracellular regulated receptor kinase 1/2 (ERK1/2) (Figure 1A) (Luo et al., 2013) and death-associated protein kinase 2 (DAPK2) in neonatal rat cardiomyocytes (Figure 1A) (Luo et al., 2013). As ERK1/2 phosphorylation and DAPK2 phosphorylation can mediate apoptosis, they may explain which H_2_-histamine receptors in the heart can induce apoptosis (Luo et al., 2013; Zeng et al., 2014). Apoptosis could be initiated by a H_2_R-mediated increase in the protein expression of calcineurin (=protein phosphatase 2B) in neonatal rat fibroblasts (Zeng et al., 2014). This calcineurin also increased proliferation in neonatal rat fibroblasts (Zeng et al., 2014). In neonatal rat fibroblasts, the stimulation of H_2_-histamine receptors by amthamine increased the translocation of the nuclear factor of activated T-cells c3 (NFATc3) to the nuclear fraction of these cells, as well as the expression of α-smooth muscle actin (αSMA) (Zeng et al., 2014). Similarly, the stimulation of H_2_-histamine receptors in neonatal rat cardiomyocytes could also increase the protein levels of the proapoptotic caspase 3 (in Western blotting), which could also contribute to H_2_R-mediated cardiac apoptosis (Figure 5) (Zeng et al., 2014). The stimulation of neonatal rat cardiomyocytes for 24 h with histamine increased the protein expression of the proapoptotic protein Bax (=homolog of Bcl-2, an apoptosis activator) and the translocation of Bax to mitochondria in these cells, where Bax may have contributed to mitochondrial-initiated apoptosis (Luo et al., 2013; Zeng et al., 2014). Moreover, H_2_-histamine receptors can lead to the release of proteins like atrial natriuretic peptide(s) (ANP) from neonatal rat cardiomyocytes (Luo et al., 2013). Whether these signal transduction pathways are used in adult hearts or even in human hearts remains an open question that should be addressed in future research.

**TABLE 6:** Signal transduction of H_2_-histamine receptors.

Signal	Species/cell type	References
cAMP↑	^1^Guinea pig Langendorff-heart, ^2^human cardiac atrium	^1^ Kukovetz et al. (1973)
		^2^ Sanders et al. (1996)
PKA activity ↑	human cardiac atrium	Sanders et al. (1996)
L-Ca^2+^-channel activity ↑	human left ventricular papillary muscle	Eckel et al. (1982)
Adenylyl cyclase- activity↑	human cardiac left and right ventricle	Bristow et al. (1982a,b)
Gi	^1^Guinea pig adult cardiomyocytes	^1^ Belevych et al. (2004)
	^2^human right cardiac atrium	^2^ Kilts et al. (2000)
Gq	rat adult cardiomyocytes	Wellner-Kienitz et al. (2003)
GIRK (GTP-binding protein coupled inwardly rectifying potassium current) ↑	rat adult cardiomyocytes	Wellner-Kienitz et al. (2003)
Bax ↑	neonatal rat cardiomyocytes	Luo et al. (2013)
		Zeng et al. (2014)
TnFα (tumor necrosis factor alpha) release ↑	adult rat heart reperfusion	Gilles et al. (2003)
ANP ↑	neonatal rat cardiomyocytes	Luo et al. (2013)
Apoptosis ↑	neonatal rat cardiomyocytes	Luo et al. (2013)
		Zeng et al. (2014)
β-MHC (beta myosin heavy chain) ↑	neonatal rat cardiomyocytes	Luo et al. (2013)
Translocation of BAX to mitochondria	neonatal rat cardiomyocytes	Luo et al. (2013)
Phosphorylation state of ERK1/2 ↑	neonatal rat cardiomyocytes	Luo et al. (2013)
Phosphorylation state of DAPK2 ↑	neonatal rat cardiomyocytes	Luo et al. (2013)
Calcineurin ↑	neonatal rat cardiac fibroblast	Zeng et al. (2014)
Translocation of NFAT (nuclear factor of activated T-cells)	neonatal rat cardiac fibroblast	Zeng et al. (2014)
α-SMA (smooth muscle actin) ↑	neonatal rat cardiac fibroblast	Zeng et al. (2014)
Cleavage caspase 3 ↑	neonatal rat cardiomyocytes	Zeng et al. (2014)

The signal transduction mechanism(s) described in the literature for H_2_-histamine receptor activation in the heart are listed. Increase (↑) and decrease (↓). First column indicates the biochemical signal in that study (third column) and the cell system and species where this study was done. Some abbreviations: ANP, atrial natriuretic peptide; Bax is a homolog of Bcl-2, and an apoptosis activator; β-MHC: beta-myosin heavy chain; calcineurin, protein phosphatase 2B or 3; DAPK2, death associated protein kinase 2; ERK, an extracellularly activated protein kinase; G_i_, pertussis toxin sensitive inhibitory GTP, binding protein; GIRK, GTP-binding protein coupled inwardly rectifying potassium current; G_q_, GTP, binding protein; PKA, cAMP-dependent protein kinase; TnFα, tumour necrosis factor alpha.

H_2_R couples not only through stimulatory G-proteins in the heart but also via inhibitory G-proteins (Figure 2) (Kilts et al., 2000; Belevych et al., 2004) and via so-called Gq proteins (Figure 2) (Wellner-Kienitz et al., 2003). Similarly, H_2_-histamine receptors couple not only to cardiac L-type Ca^2+^ channels but also to potassium channels (Figure 1A) in the sarcolemma (Wellner-Kienitz et al., 2003).

As mentioned above, published data have shown that in the human cardiac right atrium, H_2_R stimulation also increases the level of 3′, 5′-cyclic guanosine monophosphate (cGMP) (Figure 1) (Sanders et al., 1996). These authors speculated (Figure 1A) that H_2_R may be responsible for the production of nitric oxide (NO) in cardiomyocytes or in endothelial cells. This NO may lead to an increase in cGMP via the activation of guanylyl cyclase (Figures 1B, 3) (Sanders et al., 1996). The produced cGMP can inhibit the activity of phosphodiesterase III (Figures 1B, 3) (Sanders et al., 1996). This inhibition would raise cAMP levels in the cells, which would contribute to a positive inotropic effect of H_2_R in the human heart (Figure 1B, 3) (Sanders et al., 1996). This interesting hypothesis (Sanders et al., 1996) has apparently never been tested experimentally. Moreover, although it could be shown that the H_2_R increases the force of contraction in the human right and left ventricles *in vitro* (Ginsburg et al., 1980; Bristow et al., 1982b) and activates ventricular adenylyl cyclase (Bristow et al., 1982a; 1982b), to the best of our knowledge, an effect of H_2_-histamine receptors on cAMP levels or phospholamban phosphorylation in the human cardiac ventricle has never been reported, which warrants future research.

The signal transduction of H_1_R (Figure 1B) differs from the signal transduction of H_2_R in the heart. Some previous studies claimed that the positive inotropic effect of H_1_R stimulation on rabbit heart was due to an increase in IP_3_ content in the heart because H_1_R stimulation was accompanied by an increase in cardiac IP_3_ levels (Figure 1B) (Sakuma et al., 1988). However, the positive inotropic effect of H_1_R stimulation was still observed in rabbit heart in the presence of inhibitors of IP_3_-generation (Hattori et al., 1989). Thus, it might be concluded that an increase in cardiac IP_3_-content does not cause the positive inotropic effect of histamine in rabbit heart. Subsequently, it was reported that the positive inotropic effect of histamine on guinea pig atrium led via H_1_R to the tyrosine phosphorylation of regulatory cardiac proteins. This increase in the phosphorylation of the amino acid tyrosine of currently unidentified proteins of apparent molecular weights of 25, 35, 65, and 150 kDa may have caused a positive inotropic effect via the H_1_-histamine receptors, as pre-treatment with a tyrosine kinase inhibitor abolished any positive inotropic effect of histamine in guinea pig atrium (Akaishi et al., 2000). It was suggested that the tyrosine phosphorylation of, for instance, myofilaments might have led to an increase in the Ca^2+^ sensitivity of the myofilaments, which may have caused the positive inotropic effect on histamine in the left atrium of guinea pig (Akaishi et al., 2000).

## 6 Electrophysiological Effects of Cardiac Histamine Receptor Stimulation

To better understand the mechanism of the inotropic, chronotropic and pro-arrhythmogenic effects of histamine on the human heart, it is necessary to review the electrophysiological effects of histamine on the heart of laboratory animals and human surgical samples (see Table 7). The stimulation of both H_1_- and H_2_-histamine receptors can affect cardiac ionic currents. Thus, in guinea pig atrial cells, histamine enhanced the slow delayed rectifier potassium current (I_Ks_), the slow component of the repolarising current I_K_, via H_1_R and via protein kinase C (PKC) with an EC_50_-value (=half maximal effective concentration values) of 0.7 µM (Matsumoto et al., 1999). On the rapid component of I_K_, I_Kr_ (=rapid delayed rectifier potassium current), histamine exerted an inhibitory effect via H_1_-histamine receptors with an EC_50_-value of 0.3 µM in a PKC-independent manner (Matsumoto et al., 1999). The overall effect of the stimulation of H_1_-histamine receptors in atrial cardiomyocytes is the prolongation of the AP (Amerini et al., 1982; Borchard and Hafner 1986; Hattori et al., 1988b), which might result from a higher contribution of the I_Kr_ component, lower EC_50_ for I_Kr_ (inhibition) or from additional effects, such as the inhibition of I_K. ACh_ (=G-protein gated potassium channel (Tohse et al., 1995). However, the effects of H_1_R stimulation may depend on the density of I_Kr_, I_Ks_, and the cell type. The reason is that in ventricular guinea pig cardiomyocytes, a shortening of the AP was observed (Valenzuela and Zhou 1992). The stimulation of the H_2_R in guinea pig ventricular cardiomyocytes increased the repolarising current I_K_ with an EC_50_ of 38 nM via the cAMP-PKA pathway, which could explain the shortening of the AP (Yazawa and Abiko 1993).

**TABLE 7:** Electrophysiological actions of histamine in the mammalian heart.

Species	Sinus node	Atrium	AV-node	Purkinje fibers	Ventricle	References
Dog	H_2_-receptor: → positive chronotropic effect^2^, H_1_-receptor → negative chronotropic effect^2^	n.d	H_2_-receptor: → positive dromotropic effect^2^, H_1_-receptor → negative dromotropic effect^1,2^	n.d	n.d	^1^ Flacke et al. (1967)
						^2^ Hageman et al. (1979)
Sheep	n.d	n.d	n.d	H_2_-receptors: activate^3^ L-type Ca^2+^, APD (action potential duration) ↓^4^, oscillations of action potentials and DAD (delayed after-depolarisations)^1,3,4^	n.d	^3^ Mugelli et al. (1980)
						^4^ Cerbai et al. (1990)
Monkey	n.d	H_2_-receptor: Right atrium: increase in beating rate	n.d	H_2_-receptor: APD ↓	H_2_-receptor: L-type Ca^2+^ and Ca^2+^ induced arrhythmias	Hattori et al. (1983)
Man	n.d	H_2_-receptor: DAD^6^, slope of phase IV ↑^6^, spontaneous depolarisations ↑^6^, amplitude of AP^6^ ↑	n.d	n.d	H_2_-receptor: prolongation of monophasic action potentials^5^	^5^ Eckel et al. (1982)
						^6^ Levi et al. (1981)
Guinea pig	n.d	H_1_-receptor: (left atrium) AP prolonged^7^ L-type-Ca^2^-channels activated^7,9^, amplitude of AP ↑^7^ cell hyperpolarizes V_max_ (maximal velocity of the action potential) ↑^7^	H_1_-receptor: AV-inhibition until block^13,12^ faster AP V_max_ and amplitude of AP ↑	n.d	H_1_-receptor: L-type Ca^2+^ channels ↑^19^ H_2_-receptor: AP prolonged^20,21^ APD shortened^7,17^ DAD, arrhythmias^21^ L-type Ca^2+^ channels ↑^7,19^ H_1-_ and H_2_-receptors, threshold of fibrillation ↓^16^, V_max_ of AP↑ slow action potentials ↑^17,18^, idioventricular rate ↑^15^	^7^ Borchard et al. (1986)
						^8^ Vial et al. (1991)
						^9^ Kecskeméti (1978)
						^10^ Levi and Giotti (1967)
						^11^ Levi et al. (1981)
						^12^ Levi (1972)
						^13^ Capurro and Levi (1973)
						^14^ Senges et al. (1977)
						^15^ Levi and Zavecz (1979)
						^16^ Trzeciakowski and Levi (1982)
						^17^ Houki (1973)
						^18^ Inui and Imamura (1976)
						^19^ Hescheler et al. (1987)
						^20^ Muramatsu et al. (1987)
						^21^ Levi and Alloatti (1988)
Rabbit	H_2_-receptor: amplitude AP ↑^22^, maximum diastolic potential ↑^22^ steepness of AP^23^ frequency of AP ↑ ^23^ DAD^22^ L-type-Ca^2+^-channels ↑^22^ I_f_-current ↑^22^ steepness of phase IV AP ↑^23^	n.d	n.d	n.d	H_2_-receptor: APD ↓^24^ AP amplitude ↑^24^ H_1_-receptor: APD ↑^24^ AP amplitude ↑^24^	^22^ Satoh (1993)
						^23^ Levi and Giotti (1967)
						^24^ Hattori et al. (1990)
Neonatal Guinea pig atria	n.d	H_2_-receptor: APD ↑	n.d	n.d	n.d	Agata et al. (2010)

This table lists in the first column the different species from which the heart, the tissue or cardiomyocytes were taken. The second to sixth column differentiate in which region of these hearts the measurement was performed. This is to show that species- and region-specific effects of histamine exist. These species differences have to be kept in mind when one wants to translate animal data to the clinic. AP: action potential, APD ↓: shortened AP, duration. APD ↑ prolonged AP, duration. DAD: delayed afterdepolarization. I_f_: funny (fuzzy, HCN)-current = pacemaker current in the sinus node. Increase (↑) and decrease (↓). Oscillations in this table mean that abnormal spontaneous automatic deporalizations and repolarizations of monophasic action potentials were recorded in multicellular preparations. N.d. stands for none documented. V_max_: maximal velocity of the action potential.

Hageman et al. (1979) studied living adult mongrel dogs (sex not reported) anesthetized by sodium pentobarbital and ventilated by an endotracheal tube. They used 2-methylhistamine (100 µg as bolus) as a H_1_-histamine receptor agonist and 4-methylhistamine (100 µg as bolus) as a H_2_-histamine receptor agonist and applied these compounds via the sinus node artery to detect effects on the sinus node (Hageman et al.,^1979^). Similarly, the drugs were also injected into the atrioventricular node artery and surface ECGs, were recorded (Hageman et al.,^1979^). Flacke et al. (1967) used also living adult dogs (not selected by sex or breed) anesthetized by sodium pentobarbital and ventilated the lungs. They injected histamine (bolus 0.1–10 mg) and/or diphenhydramine (bolus 3 mg, as an H_1_-histamine-receptor antagonist) in the venous inflow tubing near the entrance of the right heart (Flacke et al.,^1967^). Cerbai et al. (1990) used isolated Purkinje fibers from sheep heart obtained from a slaughterhouse (age and sex not reported). These isolated Purkinje fibers were electrically stimulated and transmembrane action potentials were recorded (Cerbai et al.,^1990^). They used 1–100 µM of 4-methylhistamine or dimaprit as H_2_-histamine receptor agonists and 10 µM cimetidine as H_2_-histamine receptor antagonist (Cerbai et al.,^1990^). Likewise, Purkinje fibers were isolated from sheep hearts obtained from a slaughterhouse (age and sex not reported) electrically stimulated in the presence of low potassium ion concentrations (0.53 mM, Mugelli et al.,^1980^). Then the electrical stimulation was stopped, 10 µM histamine was added and histamine induced spontaneous oscillatory activity of the transmembrane action potentials, indicative of cardiac arrhythmias induced by histamine, were recorded (Mugelli et al.,^1980^). These effects of histamine were abolished by the H_2_-histamine receptor antagonist burimamide (20 μM, Mugelli et al.,^1980^).

Eckel et al. (1982) studied isolated electrically-stimulated human papillary muscle samples from thirteen female and four male patients, aged 5–72 years. They used 0.1 µM to 10 mM histamine or 0.1–100 µM dimaprit in the absence or presence of 10 µM cimetidine (Eckel et al.,^1982^). They studied action potential duration (APD, 90 and 20%, Eckel et al.,^1982^). Levi et al. (1981) studied right atrial preparations from patients ageing 1–65 years (sex was not reported). They measured in these samples during spontaneous activity the transmembrane action potential (Levi et al.,^1981^). They used 1 nM -100 µM histamine in the absence and presence of 3–30 µM cimetidine (Levi et al.,^1981^). Houki, 1973, studied right ventricular papillary muscle from guinea pigs (sex not given). Houki recorded transmembrane action potential at 30°C and used 0.1–100 µM histamine (Houki, 1973). Borchard et al. (1986) studied isolated electrically driven left atrial preparations or right ventricular papillary muscles from guinea pigs. They studied 1–10 µM histamine, dimaprit or impromidine in the absence and presence of 10 µM cimetidine, or 10 µM dimetindene, a H_1_-histamine receptor antagonist (Borchard et al.,^1986^). Levi and Zavecz (1979) used a modified Langendorff-set-up for isolated guinea pig hearts. They opened surgically the atrium and using a silk suture and mechanically brought about a complete atrioventricular conduction block in these hearts (Levi and Zavecz 1979). Surface ECG, from the hearts were recorded as read out (Levi and Zavecz, 1979). They injected into the aorta histamine (0.1–30 µg), 4-methylhistamine and 2-(2-thiazolyl) ethylamine, a H_1_-histamine receptor agonist at low concentrations, alone or in addition cimetidine (3 µM) or chlorpheniramine (a H_1_-histamine receptor agonist at low concentrations 1 µM) (Levi and Zavecz, 1979). Trzeciakowski and Levi (1982) used male guinea pigs of 250–300 g weight for Langendorff studies. Two needle electrodes were inserted into the ventricle of spontaneously beating guinea pig hearts to induce ventricular arrhythmias and thus establish a threshold for arrhythmogenesis (Trzeciakowski and Levi 1982). They used as agonists histamine, pyrilamine, 2-(2-thiazolyl) ethylamine, tiotidine and impromidine (0.3 nM-1 mM, Trzeciakowski and Levi, 1982). Inui and Imamura (1976) isolated papillary muscles from 350–500 g weighing guinea pigs. They measured transmembrane action potentials under physiological conditions and in the presence of 27 mM potassium cations in order to depolarize the muscle and to measure slow potentials being indicative of the action of the L-type Ca^2+^-channel (Inui and Imamura, 1976). They used histamine (0.3–30 µM), metiamide (a H_2_-histamine receptor antagonist 3 µM), and diphenhydramine (a H_1_-histamine-receptor agonist at low concentrations 10 μM, (Inui and Imamura, 1976). A similar approach as Ini and Imamura (1976) was used by Kecskeméti (1978), but on left atrial preparations from the guinea pig heart. Senges et al. (1977) studied isolated papillary muscles from the right ventricles of the guinea pigs (400–600 g). They recorded transmembrane action potentials in electrically paced preparations (Senges et al.,^1977^). They used histamine (20 µM) and 20 µM burimamide (a H_2_-histamine receptor antagonist) and brompheniramine (a H_1_-histamine receptor antagonist) (Senges et al.,^1977^).

Muramatsu et al. (1987) isolated ventricular cardiomyocytes from adult guinea pigs (sex not recorded). Thereafter, they applied the whole cell patch clamp technique to record currents through L-type Ca^2+^-channels (Muramatsu et al.,^1987^). Levi and Alloatti (1988) isolated ventricular cardiomyocytes from guinea pigs, weighing 200–300 g of either sex. These cells were used for patch clamp experiments at room temperature (Levi and Alloatti, 1988). Others used the same technique as Levi and Alloatti (1988) as but recorded at 35°C (Hescheler et al., 1987). Levi and Giotti (1967) studied isolated sinus node preparations from rabbits of either sex (1.5 kg weight). Measurements took place at 30°C and samples were beating on their own (Levi and Giotti, 1967). Satoh (1993) isolated sinus node preparations from rabbits (1.5–2 kg, sex not reported) and recorded at 36°C. Hattori et al. (1990) studied right ventricular papillary muscles from rabbits of either sex weighing 2–3 kg. In these preparations, transmembrane action potentials were recorded during electrical stimulation (Hattori et al.,^1990^). Similarly, Hattori et al. (1983) studied the heart of Japanese monkeys of either sex weighing 3–5 kg. They excised left atria, right atria, right ventricular papillary muscles, right ventricular Purkinje fibers, left and right ventricular wall strips (Hattori et al. 1983). Where necessary, samples were electrical stimulated and transmembrane action potentials were recorded using 1 µM histamine alone or in the presence of 10 µM cimetidine (Hattori et al.,^1983^).

In the past, slow APs were used as a surrogate for the Ca^2+^ carried slow inward current and for testing the effects of calcium antagonistic drugs. These slow potentials were elicited by histamine (1–10 µM) in K^+^ (20–30 mM) depolarised papillary muscles (e.g., Barbieri et al., 1991). They, however, indicated a possible direct or indirect effect of histamine on the cardiac L-type Ca^2+^ channel current (I_Ca.L_), which is in line with the finding that histamine can elicit spontaneous APls in Purkinje fibres in sheep (Cerbai et al., 1990). Accordingly, in guinea pig atrial myocytes, histamine enhanced the intracellular Ca^2+^-concentration measured by Indo-1 (a calcium indicator) fluorescence via H_1_-histamine receptors in a nifedipine-sensible way (Yoshimoto et al., 1998). Although it could be assumed that this could indicate the stimulation of I_Ca.L_, it was shown that this effect of histamine on the Ca^2+^ current was an indirect effect by prolonging the AP duration, due to the inhibition of the outward potassium current, thereby extending the time window for the influx of I_Ca.L_ and Ca^2+^ ([Ca^2+^]_i_) (Yoshimoto et al., 1998). Whole cell voltage clamp experiments showed that histamine did not directly alter I_Ca.L_ (Yoshimoto et al., 1998). However, in guinea pig ventricular cells, H_2_R stimulation enhanced the amplitude of the slow Ca^2+^-current. This effect was mimicked by GDP_ϒ_S (=in gamma position sulphur substituted guanosine triphosphate) (Hescheler et al., 1987).

Also modulated by histamine was the pacemaker current I(f) (=the hyperpolarisation-activated inward current (formerly known as I [h] and now as I [f]), which was enhanced via the stimulation of H_2_-histamine receptors in rabbit sinoatrial nodal cells (Satoh 1993). In addition, it was shown by the overexpression of H_2_-histamine receptors in rat atrial cells that histamine—in this experimental situation—inhibited I_K. ACh_ (Wellner-Kienitz et al., 2003), which was previously shown in earlier guinea pig atrial cells (Tohse et al., 1995). Thus, the increase in heart rate following histamine administration may be attributable to a combined effect that includes the stimulation of I(f), the enhancement of I_Ca.L_, the increase in [Ca^2+^]_i_ and at least partial inhibition of I_K. ACh_.

Combined, the effects of histamine on the electrophysiology of the heart depend on the amount and subtype of histamine receptors (H_1_R; H_2_R), on the density of the target channels and currents (I_Kr_; I_Ks_; I_K. ACh_; I_f_; I_Ca.L_) and the area of the heart under investigation (i.e., the sino-atrial node, atrioventricular (AV) node, the conduction system and the ventricle), which varies among species. An overview is provided in Table 7.

The results shown in Tables 4, 7 indicate that the electrophysiological effects of histamine were recorded only in species where inotropic effects of histamine were detectable. The involvement of H_2_-histamine receptors usually leads to electrophysiological effects that are opposite those of the involvement of H_1_-histamine receptors. Table 7 also shows mechanisms for the arrhythmogenic effects of histamine. The term “slow APs” indicates the effect of histamine on partially depolarised cardiac tissue when calcium cations, not sodium cations, carry the sarcolemmal current.

## 7 Comparison of the Potency of H_2_R Agonists in Inotropy and Chronotropy in Several Species

The first investigators in the field of histamine pharmacology noted a histamine-induced positive inotropic effect in the hearts of experimental mammals (Dale and Laidlaw 1910). These early researchers noted that histamine was also active in the human cardiovascular system, which paved the way for future research (Dale and Laidlaw 1910). As shown in Table 8, a positive inotropic effect or a positive chronotropic effect of histamine or its derivatives was observed in humans and in several laboratory animals. However, in some frequently used laboratory animals, histamine did not act on the force of contraction via histamine receptors but by the release of noradrenaline (Table 8) in mice (Gergs et al., 2019) and rats (Laher and McNeill 1980a). Animals such as wild-type mouse, rat and cat are not useful as model systems of the human heart. Mice with the overexpression of H_2_-histamine receptors may be a better choice (Gergs et al., 2019, 2020). They have been successfully used to predict the effects of H_2_-histamine receptor agonists or H_2_-histamine receptor antagonists on human hearts (Neumann et al., 2021b,c).

**TABLE 8:** EC_50_-values for H_2_-histamine receptor agonists on isolated cardiac preparations from various species.

Agonist	System	Species	EC_50_ -values	Effectivity, force or frequency measured	References
Histamine	right ventricular papillary muscle	ferret (male, 12–14 weeks)	5.90	Force	Hurrell et al. (1993)
Amthamine	left ventricular papillary muscle	Guinea pig (250–350 g)	6.17	full agonist force	Poli et al. (1993)
Amthamine	right atrial preparations	Guinea pig (250–350 g)	6.72	full agonist frequency	Poli et al. (1993)
Amthamine	left ventricular papillary muscle	Guinea pig (300–400 g)	6.17	force	Coruzzi et al. (1995)
Amthamine	right atrial preparations	Guinea pig (300–400 g)	6.72	frequency	Coruzzi et al. (1995)
Dimaprit	left ventricular papillary muscle	Guinea pig (male, 300–400 g)	4.88	force	Poli et al. (1993)
Dimaprit	right atrial preparations	Guinea pig (250–350 g)	5.32	full agonist frequency	Poli et al. (1993)
Dimaprit	left and right ventricle (Langendorff)	Guinea pig (female, 400–550 g)	6.2 × 10-9 mol (bolus)	force	Baumann et al. (1981b)
Dimaprit	right atrial preparations	Guinea pig (male, 350–400 g)	5.74	frequency partial agonist	Krielaart et al. (1990)
Histamine	left ventricular papillary muscle	Guinea pig (250–350 g)	5.92	force	Poli et al. (1993)
Histamine	right atrial preparations	Guinea pig (250–350 g)	6.01	frequency	Poli et al. (1993)
Histamine	right atrial preparations	Guinea pig (male)	5.95	frequency	Reinhardt et al. (1974)
Histamine	right atrial preparations	Guinea pig (male)	6.07	force	Reinhardt et al. (1974)
Histamine	left atrial preparations	Guinea pig (male)	5.90	force	Reinhardt et al. (1974)
Histamine	right atrial preparations	Guinea pig	5.85	frequency	Krielaart et al. (1990)
Histamine	left ventricular papillary muscle	Guinea pig (300–400 g)	6.30	force	Bertaccini und Coruzzi (1981)
Histamine	left atrial preparations	Guinea pig (male, 300–500 g)	5.92	force	Sakuma et al. (1988)
Histamine	ventricular adult cardiomyocytes	Guinea pig (both, 200–300 g)	8.00	L-type Ca2+-current	Levi and Alloatti (1988)
Histamine	left and right ventricle (Langendorff)	Guinea pig (female, 400–550 g)	2.4 × 10-9 mol (bolus)	force	Baumann et al. (1981b)
Histamine	neonatal left atrium	Guinea pig	5.29	force	Agata et al. (2010)
Histamine	right ventricular papillary muscle	Guinea pig (250–450 g)	6.16	force	Hattori et al. (1994)
Histamine	left ventricle (Langendorff)	Guinea pig (male, 250–300 g)	7.27	frequency	Trzeciakowski and Levi (1982)
Impromidine	left ventricle (Langendorff)	Guinea pig (male, 250–300 g)	8.30	frequency	Trzeciakowski and Levi (1982)
Impromidine	left and right ventricle (Langendorff)	Guinea pig (female, 400–550 g)	3.3 × 10-11 mol (bolus)	force	Baumann et al. (1981b)
4-Methylhistamine	right atrial preparations	Guinea pig (both, 300–500 g)	5.44	partial agonist, frequency	MacLeod and McNeill (1981)
4-Methylhistamine	left atrial preparations	Guinea pig (both, 300–500 g)	5.82	force	MacLeod and McNeill (1981)
4-Methylhistamine	left atrial preparations	Guinea pig (male, 300–400 g)	n.d. (no plateau was reached)	force	Amerini et al. (1982)
4-Methylhistamine	right ventricular strips	Guinea pig (male, 300–400 g)	n.d. (no plateau was reached)	force	Amerini et al. (1982)
4-Methylhistamine	right atrial preparations	Guinea pig (male, 300–400 g)	n.d. (no plateau was reached)	frequency	Amerini et al. (1982)
Amthamine	right atrial preparations	man	5.38	full agonist; force	Poli et al. (1993), (1994)
Amthamine	right atrial preparations	man	5.38	force	Coruzzi et al. (1995)
Dimaprit	right atrial preparations	man	4.37	full agonist; force	Poli et al. (1993), (1994)
Histamine	right and left ventricular papillary muscles	man	5.60	force	Bristow et al. (1982b)
Histamine	left ventricular papillary muscle	man (11 male, 16 female, 40–69 years)	5.64	force	Brown et al. (1986)
Histamine	left ventricular papillarly muscle	man (14 female, 4 male, 5–72 years)	5.41	force	Eckel et al. (1982)
Histamine	right atrial preparations	man (26 female 60 male, 33–75 years)	5.5	force	Zerkowski et al. (1993)
Histamine	right atrial preparations	man	5.19	force	Poli et al. (1993), (1994)
Histamine	right atrial preparations	man (both, 60–78 years)	n.d	force	Neumann et al. (2021e)
Impromidine	right atrial preparations	man	6.59	partial agonist; force	Poli et al. (1994)
Impromidine	left ventricular papillary muscle	man (12 male, 8 female)	around 5.0	partial agonist; force	English et al. (1986)
Histamine	left atrial preparations	monkey (both, 3–5 kg)	7.04	force	Hattori et al. (1983)
Histamine	right atrial preparations	monkey (both, 3–5 kg)	6.22	frequency	Hattori et al. (1983)
Histamine	right ventricular papillary muscle	monkey (both, 3–5 kg)	6.70	force	Hattori et al. (1983)
Dimaprit	left atrial preparations	mouse: H2-TG (both, 60–90 days)	6.39	force	Gergs et al. (2019), (2020)
Histamine	left atrial preparations	mouse: H2-TG (both, 60–90 days)	6.73	force	Gergs et al. (2019), (2020), (2021a)
Histamine	right ventricular papillary muscle	rabbit (both, 1.8–2.5 kg)	5.79	force	Hattori et al. (1994)
Histamine	left atrial preparations	rabbit (both, 1.8–2.5 kg)	5.53	force	Hattori et al. (1988a)
Histamine	right atrial preparations	rabbit (both, 1.8–2.5 kg)	5.47	force	Hattori et al. (1988a)
Impromidine	left atrial preparations	rabbit (both, 1.8–2.5 kg)	8.69	force	Hattori et al. (1988a)
Impromidine	right atrial preparations	rabbit (both, 1.8–2.5 kg)	8.55	force	Hattori et al. (1988a)
Histamine	neonatal rat cardiomyocytes spontaneously beating	Rat (One to 2 days old)	6.30	frequency	McCall and Lui (1986)

Here, some of the agonists at H_2_-histamine-receptor (first column) have been compared for their inotropic or chronotropic potencies (fourth column), the signal studied (force = force of contraction, frequency: beating rate: fifth column), differentiated according to region of the heart (second column) and species studied (third column) and the references is given in the last column. The table is ordered firstly by species and therein by agonist. Right atrial preparation means that in isolated preparations the force of contraction was measured in spontaneously beating preparations and the intervals between beats have been used by the authors to assess the potency of the agonist on beating rate (=frequency of beating) and used this to calculate its positive chronotropic effect. In the paper from McCall and Lui (1986), movement of the wall of neonatal rat cardiomyocytes was used to assess the beating rate under a microscope. Left atrial preparations or left ventricular preparations (usually papillary muscle sometimes strips of ventricular walls were used) indicates that in isolated preparations the force of contraction was measured in electrically stimulated preparations and the authors used force to assess the potency of the agonist to exert a positive inotropic effect. “Langendorff” in the columns means that an isolated spontaneously beating buffer perfused heart was studied (Langendorff 1895). Baumann et al. (1981b) measured with balloons the pressure in the right ventricle as well as the left ventricle in isolated spontaneously beating hearts according to Langendorff (1895). In brackets, in the third columns “both”, “male” or female” refer to gender of patients or animals and “g” to body weight in grams in animals. If data are lacking in brackets, no data to gender or age or weight were found in the original publication. n.d. indicates that the value was not documented, for instance, because not enough agonist in the concentration response curve was used to reach saturation of the effects (=no plateau reached). If for drug that acts on a histamine receptor, there is added “full agonist” or “partial agonist”: this means that in that study the drug was as effective as histamine (full agonist) or less effective than histamine (partial agonist).

## 8 Adenylyl Cyclase-dependent Signalling of Histamine

The involvement of adenylyl cyclase in the positive inotropic effect of histamine (Figures 1A,B) was initially suggested by Pöch and Kukovetz (1967) and later tested directly by measuring cardiac adenylyl cyclase activity and by measuring cAMP levels in isolated freeze-clamped cardiac preparations. It was tested indirectly by inhibiting the degradation of cAMP by treatment of cardiac preparations with phosphodiesterase inhibitors (Kukovetz et al., 1973). Klein and Levey (1971) were the first to report that histamine could stimulate the activity of adenylyl cyclase in broken cell preparations from guinea pig hearts, one human heart and cat hearts. The data on kitten cardiac membranes (Klein and Levey 1971) are probably doubtful, as the cat has no functional H_2_-histamine receptors (Table 4) (Laher and McNeil 1980c). Because these early researchers had no H_2_R antagonist at their disposal, they could only block the activity of adenylyl cyclases with very high concentrations of promethazine, which were at lower concentrations a H_1_R antagonist but at higher concentrations in an H_2_R antagonist (Klein and Levey 1971). In contrast, other researchers reported that the histamine-induced stimulation of guinea pig membrane-bound adenylyl cyclase was not inhibitable by promethazine at concentrations that were specific for H_1_-histamine receptors (McNeill and Muschek 1972), that is, in concentrations that were so high that, as we now know, promethazine also blocked H_2_-histamine receptors. However, they later obtained samples of burimamide (the first reported H_2_R antagonist) (Black et al., 1972). They showed that burimamide antagonised the histamine-stimulated activity of adenylyl cyclase in guinea pig cardiac membranes (Verma and McNeill 1974). Over time, it became apparent that histamine increased the potency and effectiveness of the activity of guinea pig cardiac adenylyl cyclases if guanylnucleotides were added to the incubation medium, which was the first indication of the involvement of GTP-binding proteins in this process (Figures 1A,B). Under these experimental conditions, dimaprit, N^α^-methylhistamine, and 4-methylhistamine were partial agonists, and PEA (now regarded as a H_1_R agonist) was ineffective in increasing the activity of adenylyl cyclase in guinea pig cardiac membranes (Johnson et al., 1979) (Table 1) in agonists often used in cardiac pharmacology. The stimulatory effects of histamine on guinea pig adenylyl cyclase in cardiac membranes were also antagonised by clinically relevant antagonists, such as cimetidine (see Table 2 for a list of commonly used antagonists) and were thus regarded as H_2_R-mediated (Johnson et al., 1979; Kanof and Greengard 1979). However, other cardiovascular drugs, such as clonidine, stimulated cardiac adenylyl cyclase via H_2_-histamine receptors (Kanof and Greengard 1979). Clonidine is currently used as an antihypertensive agent because of its stimulatory action on central α_2_-adrenoceptors. Whether the stimulatory effect of clonidine is species specific is unclear. Therefore, future research should be conducted to determine whether clonidine also stimulates human H_2_-histamine receptors in cardiac preparations, which is currently unknown, but it might be clinically relevant.

Another potentially relevant antagonistic action in cardiac H_2_-histamine receptors has been known for many years. The stimulatory effects of histamine on the activity of adenylyl cyclase in guinea pig ventricular preparations were inhibited in a competitive fashion by antidepressant and neuroleptic drugs (Kanof and Greengard 1979) (Table 2, lower part). In isolated guinea pig Langendorff-perfused hearts, high concentrations of promethazine reduced histamine-induced increases in contractility and reduced histamine-stimulated cardiac cAMP content (McNeill and Verma 1974a), extending the biochemical data to functional data.

A study on adenylyl cyclases in human ventricles that were obtained during surgery from failing and non-failing human hearts revealed findings similar to guinea pig cardiac adenylyl cyclases. Histamine concentration dependently increased adenylyl cyclase activity, which was more effective in the presence of a non-hydrolysable GTP derivative, suggesting the involvement of G-proteins (Figure 1A) in the human heart as well (Bristow et al., 1982b). Impromidine and dimaprit (Table 1) were less effective than histamine in increasing adenylyl cyclase activity in membranes of human ventricles (Bristow et al., 1982b). However, the stimulatory effects of histamine on adenylyl cyclase activity in human ventricle membranes were cimetidine sensitive and thus were probably H_2_R-mediated (Bristow et al., 1982b). Other studies, in contrast, found that dimaprit and impromidine were as effective as histamine in stimulating the activity of adenylyl cyclase in membranes prepared from human papillary muscles (Baumann et al., 1981b). However, Bristow et al. (1982b) data were in line with functional data. In performing contraction experiments in isolated human left ventricular preparations, they found that impromidine was less effective than histamine. Moreover, impromidine antagonised the positive inotropic effects of histamine; thus, impromidine acted as a partial agonist of cardiac human H_2_-histamine receptors (English et al., 1986; Felix et al., 1995).

Based on the foregoing studies on promethazine in the heart and adenylyl cyclases isolated in guinea pig brain, drugs used in psychiatry were noted to inhibit the histamine-stimulated activity of cerebral adenylyl cyclases. Notably, amitriptyline and nortriptyline, doxepin, haloperidol, clozapine, chlorpromazine, thioridazine, and mianserin inhibited the histamine-stimulated activity of cerebral adenylyl cyclases (Green and Maayani 1977; Kanof and Greengard 1978; Dam Trung Tuong et al., 1980). The authors speculated that the inhibition of brain H_2_-histamine receptors might underlie the clinical effects of these drugs (Green and Maayani 1977; Kanof and Greengard 1978; Dam Trung Tuong et al., 1980). However, that view has been disputed (Kanba and Richelson 1983), and it is currently not the preferred explanation of the clinical effects of these psychiatric drugs. However, such data indicate that these psychiatric drugs could clinically interfere with cardiac H_2_-histamine receptors. Based on these reports, amitriptyline was recently shown to antagonise the effects of histamine on the force of contraction in isolated atrial preparations from human hearts, indicating that old data on psychiatric drugs are still clinically relevant, and they have not been considered seriously in the past (Neumann et al., 2021b).

Early data showed that histamine increased the force of contraction and the beating rate in prenatal whole human heart, right atrium, or paced ventricular preparations (Wollemann and Papp 1979). These inotropic data on human prenatal hearts were accompanied by measuring the histamine-stimulated activity of adenylyl cyclase in foetal human hearts, which was antagonised by cimetidine and therefore H_2_R-mediated (Wollemann and Papp 1979).

## 9 Histamine and cAMP in the Heart: Age- and Species-dependent Presence of Cardiac Histamine Receptors

It is likely that the first reports that histamine increased the cAMP content in whole heart were based on isolated spontaneously beating guinea pig heart (Kukovetz et al., 1973). They reported that theophylline could potentiate the positive inotropic effects of histamine in Langendorff-perfused guinea pig hearts. Theophylline was used as a phosphodiesterase (PDE) inhibitor (Kukovetz et al., 1973). Furthermore, they reported that in rapidly frozen isolated guinea pig hearts, the positive inotropic effect of histamine was accompanied and even pre-ceded by increases in cAMP content (Kukovetz et al., 1973). Data on the use of subtype-specific PDE inhibitors, such as EHNA (=erythro-9-(2-hydroxy-3-nonyl) adenine) for PDE II, cilostamide for PDE III and rolipram for PDE IV have been confirmed and extended to left atrial preparations of mice overexpressing human H_2_-histamine receptors (Neumann et al., 2021c). In mice, PDE II and IV were found to be particularly relevant for degrading cAMP formed by the stimulation of H_2_-histamine receptors, which is not necessarily true in the human heart, but it needs to be elucidated (Neumann et al., 2021c). Kukovetz et al. (1973) data were confirmed and extended by other researchers who blocked the histamine-induced increase in cAMP content using burimamide (the first H_2_R antagonist, Table 2) in contracting and rapidly frozen isolated guinea pig cardiac preparations (McNeill and Verma 1974b).

Other researchers included papaverine in their atrial preparations (Reinhardt et al., 1977). They used papaverine as a PDE inhibitor (Reinhardt et al., 1977). Papaverine shifted the effect of histamine on the force of contraction and cAMP content in guinea pig papillary muscles to lower concentrations of histamine (Reinhardt et al., 1977). Their findings also suggested that H_2_-histamine receptors are coupled with the generation of cAMP, at least in guinea pig papillary muscle (Reinhardt et al., 1977). Similarly, the positive chronotropic effect of histamine in spontaneously beating guinea pig right atrial preparations could be potentiated by papaverine (Reinhardt et al., 1977). This result suggested that cAMP was formed in the sinus node, which caused an increase in the beating rate of isolated right atrial preparations from guinea pigs (Reinhardt et al., 1977). In contrast to the results shown in the guinea pig ventricle, histamine did not increase cAMP content in isolated electrically stimulated preparations from the left atrial in guinea pigs. Moreover, the inotropic effect of histamine in isolated electrically stimulated preparations from the left atrial of guinea pigs was neither potentiated by papaverine (Reinhardt et al., 1977) nor antagonised by burimamide (Verma and McNeill 1977). These results suggest that H_2_-histamine receptors and cAMP were not involved in the effect of histamine in the guinea pig left atrial preparations but only in the guinea pig right atrium preparations (Verma and McNeill 1977). These findings are summarised in Table 4. However, the positive inotropic effect of histamine in the left atrium of guinea pigs was antagonised by the H_1_R antagonist mepyramine (Wilson and Broadley 1980), which provided evidence that the positive inotropic effects of histamine in the left atrium of guinea pigs are H_1_R-mediated. Thus, histamine can use different receptors and different second messengers in different regions of the mammalian heart (see *Brief Notes on H2R Biochemistry* and Table 4 for synopses of these findings).

Early indirect evidence suggested that the positive inotropic effect of H_2_R stimulation was mediated by the activation of L-type Ca^2+^ channels, but not the positive inotropic effect of H_1_-histamine receptors. For instance, in isolated guinea pig left atria that contained only H_1_-histamine receptors (Table 4), under potassium-induced depolarised conditions, histamine was unable to increase the force of contraction, whereas in isolated right ventricular guinea pig muscle that contained both H_1_- and H_2_-histamine receptors (Table 4), histamine elicited an increase in the force of contraction (Hattori and Kanno 1985). These findings can be explained as follows: under partial depolarisation with high potassium, the L-type Ca^2+^ channel is activated by cAMP-increasing pathways, such as the pathway initiated by H_2_-histamine receptors. However, pathways that do not use cAMP, such as H_1_R, are unable to activate the L-type Ca^2+^ channel; thus, under these conditions, they cannot generate force. The same mechanism was also measured in the isolated electrically stimulated left atrium of H_2_R overexpressing mice, where histamine elicited an increase in force under potassium depolarisation (Gergs et al., 2021a). These findings support the notion that in guinea pig atria, H_2_R stimulation increases the force of contraction by first opening L-type Ca ^2+^ channels, which leads to an increase in cytosolic free Ca ^2+^, thereby finally increasing force (Figures 1A,B) (Gergs et al., 2021a).

Regarding time parameters, H_1_R stimulation by applying the H_1_R agonist PEA in the additional presence of the H_2_R antagonist cimetidine increased the time to peak tension and the relaxation time in isolated guinea pig right ventricular strips. The positive inotropic effect was more pronounced at a stimulation rate of 1 Hz than at higher stimulation rates (Mantelli et al., 1982). In contrast, the H_2_R-mediated effect elicited by 4-methylhistamine led to the shortening of mechanical contraction parameters (Mantelli et al., 1982). Moreover, 4-methylhistamine was able to elicit a contraction in potassium depolarised isolated guinea pig right ventricular strips, which again suggested Ca^2+^ channel activation by H_2_-histamine receptors but not by H_1_-histamine receptors (Mantelli et al., 1982).

### 9.1 Age-dependent Histamine Effects

Histamine also increased cAMP content and augmented contractility (i.e., increased the amplitude of contraction and shortened both time to peak and time of relaxation) in isolated foetal mammalian cardiomyocytes in rats, which was initially reported in spontaneously beating neonatal rat cardiomyocytes (Warbanow and Wollenberger 1979). Several years later, a full-length paper (Tables 4, 8) (McCall and Lui 1986) confirmed these data in neonatal rat cardiomyocytes and extended them by showing that the positive chronotropic effects (cell length was used to obtain data for heart beating rates, but inotropy was not reported) and the cAMP-increasing effects of histamine in cell cultures of neonatal rat cardiomyocytes were antagonised by cimetidine but not by diphenhydramine and hence were H_2_R-mediated (McCall and Lui 1986). These data showed that the function of histamine in rat heart is age-related: there was an H_2_R-mediated effect in neonatal ventricular but not in adult ventricular cardiomyocytes. These data challenged the comparability of previous studies on histamine effects in neonatal rats and adult rats. We argue that the effects of histamine on cell culture work in neonatal rats, such as measuring signal transduction, cannot be translated into results in adult rats or humans without further control experiments. Similarly, isolated foetal guinea pig ventricular cardiomyocytes in culture showed an increase in contractility due to the application of histamine via H_2_-histamine receptors (Warbanow and Wollenberger 1979). Based on the data on rats, it could be predicted that one could measure a positive inotropic effect of histamine also on foetal or neonatal mouse cardiomyocytes, which rapidly vanished during the maturation of the mouse heart. Such age-related data could easily be generated, but they are currently unavailable.

Moreover, positive contractile effects of histamine and dimaprit were reported in isolated electrically stimulated adult cardiomyocytes in transgenic mice with the cardiac overexpression of H_2_-histamine receptors but not from wild-type mice. In the same adult cardiomyocytes, histamine increased the level of free cytosolic Ca^2+^. These effects were antagonised by cimetidine (Gergs et al., 2019) (Figure 1A; Tables 4, 8). We draw attention to the fact that in adult rat hearts, histamine does not stimulate histamine receptors; it releases only noradrenaline, which then increases the force of contraction (Table 4) (Laher and McNeill 1980a). In other words, in rats, H_2_R-mediated positive inotropic effects are present only in neonatal rat cells. Based on the results of Northern blots and Western blots, the receptors are still biochemically present in adult rat hearts (Matsuda et al., 2004), but they are inotropically inactive. Either they are present only in non-cardiomyocytes in adult rat hearts or they are present in cardiomyocytes themselves. In either case, the H_2_-histamine receptors in cardiomyocytes do not couple with adenylyl cyclase, or the local PDE activity is exceedingly high, which is currently unknown. The evolutionary advantage of this process in the rat heart remains an enigma. In principle, age not only leads to the loss of the histamine effect in the heart, as in the rat. Age can also alter the use of histamine receptor subtypes, which has been reported, for example, in guinea pigs. In isolated electrically stimulated right ventricular preparations from neonatal hearts, the positive inotropic effect of histamine was antagonised by the H_2_R antagonist cimetidine (10 µM) but not by the H_1_R antagonist chlorpheniramine (1 µM) (Shigenobu et al., 1980). In contrast, in isolated electrically stimulated right ventricular preparations from adult guinea pigs (300–500 g, older than 10 days), the positive inotropic effect of histamine was only slightly antagonised by the H_2_R antagonist cimetidine (10 µM). However, it was antagonised mainly by the H_1_R antagonist chlorpheniramine (1 µM) (Shigenobu et al., 1980). A different situation was found in the left atrium of guinea pigs. In isolated electrically stimulated left atrial preparations from neonatal guinea pigs, the positive inotropic effect of histamine was antagonised by the H_2_R antagonist ranitidine (10 µM), but it was not antagonised by the H_1_R antagonist chlorpheniramine (0.3 µM) or by the H_3_R antagonist thioperamide (1 µM) (Agata et al., 2010). This result suggests that in the right ventricle and left atrium of guinea pig, H_1_R gains a main inotropic role postnatally. The situation is different in the human heart: H_2_-histamine receptors are inotropically active in foetal, newborn and adult hearts (Papp and Resch 1975). The first results of a contractile response to histamine in human hearts were obtained in isolated foetal human hearts (Papp and Resch 1975). In the early foetal stage, histamine increased only the beating rate of isolated human hearts. Subsequently, in the gestational period of the foetus, effects of histamine on force in isolated atrium and ventricle were noted, which could be antagonised by burimamide or metiamide (Papp and Resch 1975). The positive inotropic effect and positive chronotropic effect of histamine increased after birth, which were classified as H_2_R-mediated (Papp and Resch 1975). In mid-foetal life, they showed that histamine decreased the rate of depolarisation and delayed atrioventricular conduction, which, based on the findings in guinea pigs, might suggest the action of H_1_-histamine receptors (Papp and Resch 1975). In severely damaged adult human hearts obtained from transplantation recipients, it was similarly noted that histamine was as potent and perhaps as effective in muscle samples drawn from the right or left atrium or from the right or left ventricle. All effects of histamine could be antagonised by cimetidine; they were regarded as being H_2_R-mediated (Ginsburg et al., 1980). In porcine heart, histamine acted only via H_2_-histamine receptors in isolated paced porcine right atrial muscle strips. Here, histamine was less potent but more effective than noradrenaline, whereas in isolated paced muscle strips from porcine left ventricle, histamine acted only via H_1_-histamine receptors. It was also less potent and less efficacious in increasing the force of contraction compared with noradrenaline (Du et al., 1993) (Table 4). As in the left guinea pig atrium, in the porcine ventricle as well as in the left atrium, the initial positive inotropic effect of histamine was followed by a negative inotropic effect that could be abrogated by the H_1_R blocker mepyramine (Du et al., 1993). This transient negative inotropic effect of histamine was also seen in three quarters of human ventricular and atrial preparations; it was not blocked by cimetidine and thus was not H_2_R-mediated (Du et al., 1993). In these series of experiments, noradrenaline was more potent than histamine in the human atrium and ventricle, but it was as efficacious as histamine in augmenting the force of contraction (Du et al., 1993). In isolated porcine atrium, the positive inotropic effect of histamine was H_2_R-mediated because the effect was blocked by cimetidine, whereas in the isolated porcine ventricle, the positive inotropic effects were not antagonised by cimetidine but by mepyramine and were thus H_1_R-mediated (Du et al., 1993).

## 10 Histamine Receptors in Human Heart

In samples of human left or right ventricular papillary muscles obtained during open heart surgery in non-failing hearts with mitral valve lesions, histamine and dimaprit (dimaprit being less potent than histamine) exerted concentration-dependent positive inotropic effects that were accompanied by a reduction in time to peak tension and time of relaxation (Eckel et al., 1982) (Tables 4, 8). Similar changes in the time parameters of contraction were later reported in transgenic mice with the cardiac overexpression of human H_2_-histamine receptors (Gergs et al., 2019). These contractile effects were antagonised by cimetidine but not by propranolol, suggesting the involvement of H_2_-histamine receptors (Eckel et al., 1982). Noradrenaline was more potent and effective than histamine in increasing the force of contraction (Eckel et al., 1982). These contractile data were later confirmed qualitatively by other researchers in isolated muscle strips from human ventricles (e.g., Du et al., 1993). However, in their studies, noradrenaline was shown to be as effective as histamine, a discrepancy that likely resulted from the fact that they used non-failing human hearts in their contraction study (Du et al., 1993). In spontaneously beating human right atrial pectinate preparations, histamine exerted a concentration-dependent positive chronotropic and inotropic effect (Guo et al., 1984) (Tables 4, 8, Figure 1). In the additional presence of cimetidine (or ranitidine in therapeutically relevant concentrations), increasing concentrations of histamine first decreased the force of contraction, and at higher histamine concentrations, increased the force of contraction (Guo et al., 1984). This result was interpreted as a transient negative inotropic effect because low concentrations of histamine-stimulated H_1_-histamine receptors that had an innate negative inotropic effect (Guo et al., 1984 (Figure 1B). In line with that hypothesis, histamine was more potent in the presence of the H_1_R antagonist pyrilamine than when only histamine was given. The inotropic effects of histamine were not due to the release of noradrenaline and the subsequent stimulation of β-adrenoceptors, because pindolol (an unselective β_1_-and β_2_-adrenoceptor antagonist) did not affect the contractile effects of histamine (Guo et al., 1984). The negative inotropic effect of the mixed H_1_R and H_2_R agonist 2-(2-thiazolyl)-ethylamine (ThEA) in the presence of cimetidine was more pronounced than the negative inotropic effect of histamine, which supported a negative inotropic effect of H_1_R stimulation (Guo et al., 1984). Moreover, in spontaneously beating musculi pectinati in the human right atrium, the H_1_R antagonist pyrilamine increased the concentration-dependent positive chronotropic effect of histamine (Genovese et al., 1988). This result was interpreted as evidence for a H_1_R-mediated negative chronotropic effect on the beating rate of the human heart (Genovese et al., 1988) (Figure 3). This conclusion was supported by the observation that the efficacy of A_1_-adenosine receptor stimulation or M_2_-muscarinic receptor stimulation to reduce the positive chronotropic effect of histamine was attenuated by the addition of pyrilamine (Genovese et al., 1988). For anatomical reasons, the effects of histamine on the human sinus node, the physiological pacemaker, were not investigated in that study. Therefore, the role of H_1_-histamine receptors compared with H_2_-histamine receptors in the human sinus node requires *in vitro* research. The authors were concerned that the inotropic effects of histamine on these preparations could have been indirect because in the human atrium, an increase in beating rate (even without receptor activation) leads to an increase in the force of contraction. Hence, the authors repeated their experiments using paced right atrial muscle strips and obtained qualitatively similar results (Guo et al., 1984), which indicated the direct negative inotropic effect of H_1_-histamine receptors.

When H_3_-histamine receptors and H_4_-histamine receptors were cloned or identified, respectively, it became possible to develop specific agonists and specific antagonists for H_3_- and H_4_-histamine receptors. It then became feasible to study both receptors in fine detail, which led to the reclassification of hitherto known H_1_R and H_2_R agonists, some of which were found to be good agonists or antagonists of H_3_-histamine receptors and H_4_-histamine receptors (Panula et al., 2015). Hence, some older studies in the literature may require new interpretations concerning histamine receptor specificity. Here, we address a controversy regarding the positive inotropic effects (Sanders et al., 1996) and negative inotropic effects (Guo et al., 1984) of histamine, which were observed in isolated right atrial or left atrial preparations of the human heart obtained during cardiac surgery. The contrasting findings from the two well-regarded groups are difficult to reconcile. The fact that in both studies, human atrial samples were obtained during surgery makes it difficult to identify the physiological functions of histamine in the atrium in healthy subjects. At least two publications reported a H_1_R-mediated effect in the human atrium: Guo et al. (1984) and Genovese et al. (1988). These effects might have resulted from the inhibition of the activity of cardiac adenylyl cyclase. Our laboratories have recently generated a transgenic mouse with the heart-specific overexpression of human H_1_R, which should help us to see here more clearly what the role of H_1_R in cardiac myocytes is. The positive inotropic effect of H_1_R stimulation on the human heart (Figure 1B, Sanders et al., 1996) was tentatively explained as follows: H_1_-histamine receptors residing in non-muscle cells or muscle cells generated NO, which was diffused in the cell or neighbouring cells, where it stimulated soluble guanylate cyclase, generating cGMP (which they measured as increased). This cGMP inhibited phosphodiesterase III, and thus cAMP levels increased, generating more force (Figure 1B) (Sanders et al., 1996). Other researchers argued that the positive inotropic effect, such as in rabbit heart and potentially in human heart, of H_1_R stimulation might be due to the coupling to phospholipase C (PLC) and the generation of IP_3_, which then binds to IP_3_-receptors in the sarcoplasmic reticulum (SR). Subsequently, cytosolic Ca^2+^ increases, and thus force increases, which Sakuma et al. (1988) showed in rabbit atrium (Figures 1A,B). Other researchers claimed that not PLC but tyrosine phosphorylation is involved. Thus, the activation of tyrosine kinases or the inhibition of tyrosine phosphatases should be involved (Akaishi et al., 2000).

However, convincing data have shown that histamine exerts a positive inotropic effect in human right and left atrial preparations obtained from prospective organ donors (Ginsburg et al., 1980; Kaliner et al., 1981). However, the possible involvement of H_1_-histamine receptors has not been extensively examined. Even non-failing donor hearts underwent drug treatment before and during cardiac explantation surgery, which might have altered the cardiac effects of histamine to some extent and might have contributed to conflicting contractile data on the role of H_1_-histamine receptors. For instance, data have shown that PDE inhibitors used to treat asthmatics or heart failure patients in desperate need potentiated the contractile function of H_2_R stimulation (Pöch and Kukovetz 1967; Neumann et al., 2021c). It cannot be excluded that such drugs have been taken by some patients. Therefore, data on healthy volunteers subjected to invasive cardiac catheterisation are of special value. Moreover, currently they are probably the best proof that histamine exerts stimulatory contractile effects on healthy human cardiac ventricles *in vivo* (Vigorito et al., 1983; 1986a; 1986b).

How does the efficacy of histamine compare with other inotropic interventions? In other words, how relevant is histamine in the human heart? These questions are relevant because histamine is of equal potency but of double efficacy compared with serotonin (acting via 5-HT_4_ receptors). Histamine also has 75% of the efficacy of maximum β-adrenergic stimulation (Zerkowski et al., 1993). In the human ventricle, histamine might be less important than in the human atrium because the maximum positive inotropic effect of histamine (i.e., its efficacy) is only half of that in the human atrium (Zerkowski et al., 1993). This finding is in contrast to earlier research on human cardiac explants, which showed that the positive inotropic effect of histamine on the left ventricle and right atrium were superimposable (Ginsburg et al., 1980). These differences might have been due to unreported differences in clinical data on patients, such as time from operating theatre to laboratory, slight differences in the preparation of buffer composition, age and gender, or drug therapy. Moreover, in the human atrium, some effects of histamine were propranolol-sensitive and thus probably due to a release of noradrenaline from cardiac storage sites (Ginsburg et al., 1980). The finding that at high single doses, histamine might release cardiac noradrenaline and thence indirectly increase force is not without precedence (Table 4). Thus, in the next section, we return to the animal model.

### 10.1 The Animal Model

In cat and probably in mouse and rat, any histamine effects on contractility are indirect: histamine releases noradrenaline. Currently, as shown in Tables 4, 8, rabbits and guinea pigs are used when a model of histamine in the human heart is sought. Guinea pigs have the disadvantage that the positive inotropic effect on the left atrium is only H_1_R-mediated. In rabbits, the ventricular effects are also mainly H_1_R-mediated. However, a mouse model was found to express functional human H_2_-histamine receptors in all regions of the heart (Gergs et al., 2019, 2020). This model has enabled research on the function of human H_2_R in the left atrium and the ventricles. However, mice do not express functional human H_1_-histamine receptors. Moreover, the human coronary system is better studied in pigs or in guinea pigs than in mice because of its greater similarity to the human coronary system. Nevertheless, mice have advantages because they are somewhat easier to keep and breed. Moreover, they could be crossbred with KO mice or other transgenic mice to study in detail cardiac signal transduction in the heart (Schwarzer et al., 2019; Gergs et al., 2020; Neumann et al., 2021d). Other approaches have also been successfully used. For example, the overexpression of H_2_-histamine receptors in rat cardiomyocytes using gene transfection was used to study the signal transduction of human H_2_R in detail (Wellner-Kienitz et al., 2003); however, mechanical function was not assessed in their study. Mice with KO of all histamine receptors are available from commercial suppliers (Neumann et al., 2014). However, they are not used frequently in cardiac research, as mice probably have no H_1_- or H_2_-histamine receptors that affect cardiac contraction (Gergs et al., 2019, 2020). However, the positive inotropic effect of histamine on the guinea pig left atrium cannot be solely explained by effects on ion currents because the maximum positive inotropic effects of histamine and isoprenaline in the left atrium of guinea pigs are similar, whereas the maximum increase in cytosolic Ca^2+^ in the atrial cardiomyocytes of guinea pigs to histamine was 50% of the maximum increase of cytosolic Ca^2+,^ which was due to isoprenaline (Yoshimoto et al., 1998). Hence, it was suggested that H_1_R (which is active in the guinea pig left atrium) stimulation might sensitise myofilaments to Ca^2+^ (Yoshimoto et al., 1998). Regrettably, this work has apparently not been continued. Therefore, it would be informative to know the identity of the four proteins in which the tyrosine phosphorylation state was found to be enhanced. It could be hypothesised that they are located in the myofilaments. Moreover, it would be interesting to know how their phosphorylation state directly alters their Ca^2+^ sensitivity or whether further signalling steps are involved. Similar findings in the human atrium were reported in a study based on the use of dimparit in addition to histamine and noradrenaline. Histamine was less potent than noradrenaline, but it was more potent than dimparit. All three drugs were of equal efficacy regarding their positive inotropic effects (Gristwood et al., 1980).

## 11 Roles of Histamine and Histamine Receptors in Cardiac Disease

Figures 3–5 are referred to in Roles of Histamine and Histamine Receptors in Cardiac Disease.

### 11.1 Histamine-Induced Arrhythmias

Histamine-induced arrhythmia was observed early in the surface electrocardiographies (ECG) of patients (Schenk 1921). Intravenous injections of histamine led to tachycardia in patients (Weiss et al., 1932). In dogs, the injection of histamine led to I-, II-, and III-degree AV-block in surface ECG (Hashimoto 1925). Histamine injection in dogs can also lead to ventricular arrhythmias (Flacke et al., 1967). In guinea pigs, histamine exerted negative dromotropic effects via H_1_-histamine receptors (Levi and Kuye 1974). It must be considered that, at least in mice, H_3_-histamine receptors are also involved in cardiac arrhythmias. Reperfusion arrhythmias occurred less frequently in H_3_R KO mice (Koyama et al., 2003a, b). This effect was indirect, as H_3_R stimulation would impair cardiac release of noradrenaline from cardiac ganglia. The effect was blunted in H_3_R KO mice, and thus fewer arrhythmias occurred (Koyama et al., 2003a, b). In an organ bath, histamine induced a positive chronotropic effect and occasional arrhythmias in trabeculae isolated from a human heart. These effects were cimetidine sensitive and thus were regarded as H_2_R-mediated (Ginsburg et al., 1980; Levi et al., 1981). It is well known that reperfusion of the heart leads to a release of histamine from the heart, which Davani et al. (2002) showed in rat hearts. The released histamine, which was partially derived from cardiac mast cells, contributed to reperfusion arrhythmias (Davani et al., 2002). However, rat hearts do not possess functional H_2_-histamine receptors. After a myocardial infarction, histamine is released, at least in part, from mast cells in the myocardium (Pierpaoli et al., 2003). Indeed, the extent of the increase in histamine in the plasma in dogs after coronary occlusion was correlated with the severity of the arrhythmias, which Wolff and Levi (1988) showed in their review.

Interestingly, *in vivo*, central and peripheral (Wolff and Levi 1986) sympathetic mechanisms contributed to histamine-induced cardiac arrhythmias; an increase in the beating rate in the heart led to the increased release of histamine isolated from guinea pig (Gross et al., 1984) or mouse heart (He et al., 2012). This release also occurred in mast-cell-deficient mice (He et al., 2012). Histamine release in cardiac ischaemia did not occur in histidine decarboxylase (HDC) KO mice, and few arrhythmias occurred (He et al., 2012). In perfused wild-type (WT) mouse hearts, ischaemia-induced arrhythmias could not be stopped by perfusion with famotidine or atenolol alone, but by their combined application (He et al., 2012).

In animal models (mainly guinea pigs) of allergic shock, histamine levels increased, which was accompanied by many forms of cardiac arrhythmias, such as sinus arrhythmias, junctional extrasystoles, AV-block, ventricular ectopy and premature beats, tachycardia, and ventricular fibrillation (Capurro and Levi 1973). Animal experiments have suggested that arrhythmias in septic shock might be treated not by H_2_R blockers alone but only in combination with H_1_R blockers (Wolff and Levi 1986; Felix et al., 1991b). Other researchers reported good anti-arrhythmic effects of H_2_R antagonists in animal models (Frommeyer et al., 2017).

Isolated spontaneously beating right atrial strips of musculi pectinati from patients were studied in an organ bath. Histamine induced arrhythmias that were both verapamil-sensitive and cimetidine sensitive (Levi et al., 1981). This result might indicate the involvement of H_2_-histamine receptors and L-type Ca^2+^ channels in histamine-induced supraventricular arrhythmias in the human heart (Levi et al., 1981). Other researchers found in paced right atrial human preparations that both dimaprit and histamine induced arrhythmias (Gristwood et al., 1980). In electrically driven muscle strips isolated from the right human atrium, Sanders et al. (1996) reported low beating rate histamine-induced arrhythmias, which were blocked by famotidine, but not by mepyramine, and thus were apparently H_2_R-mediated. In a transgenic mouse model, the overexpression of H_2_-histamine receptors per se led to a significant increase in the incidence of supraventricular and ventricular arrhythmias (Gergs et al., 2021a). This incidence was further increased by the addition of histamine (the physiological ligand) or dimaprit, which did not activate H_1_- but, in this context, it activated H_2_-histamine receptors (Gergs et al., 2021a). Hence, it might be useful to determine whether the expression of H_2_-histamine receptors is elevated in the hearts of patients suffering, for instance, from atrial fibrillation. As atrial thrombi would release histamine, H_2_R stimulation would both initiate and maintain atrial fibrillation. However, this function is under speculation at present.

Interestingly, in patients, the higher the plasma level of histamine, the higher the incidence of atrial fibrillation (Layritz et al., 2014). This is positive evidence that histamine might be a legitimate target for anti-arrhythmic therapy in future clinical trials. It has been reported that drinking red wine increases the incidence of arrhythmias. This has been suggested as due to either high histamine levels in some brands of wine and/or ethanol inhibiting the enzymes responsible for the degradation of histamine in the intestine or the heart, such as diamine oxidase (DAO) (Liang et al., 2012). In patients with allergies to some foods (e.g., kiwi) or to foods that contain large amounts of histamine (e.g., cheese and fish), an increased incidence of cardiac arrhythmias was noted (Rojas-Perez-Ezquerra et al., 2017).

Based on the literature reviewed above, a high rate of the production of histamine in patients is expected to lead to arrhythmias. Mast cells produce large amounts of histamine. A rare example of a histamine-producing disease is mastocytosis, which affects mast cell production. Patients suffering from mastocytosis show increased amounts of mast cells in the skin and/or internal organs. The histamine may reach cardiomyocytes via the bloodstream, where it may stimulate H_2_-histamine receptors. Indeed, patients affected by mastocytosis, including adults and children, have an increased incidence of arrhythmias (Rohr et al., 2005; Shaffer et al., 2006).

Histamine in plasma can directly cause arrhythmias via H_2_-histamine receptors on cardiomyocytes. It is known that histamine can also indirectly cause arrhythmias. Histamine does not need to reach cardiomyocytes. If histamine leads to a decrease in coronary perfusion, arrhythmias may result. Indeed, the histamine-induced constriction of coronary arteries is known to lead to arrhythmias. A case report showed that one patient with Quincke oedema, which also leads to high tissue and blood levels of histamine, developed coronary constriction, ST-elevation and arrhythmias (Weber et al., 1982).

Furthermore, terfenadine and astemizole (H_1_R antagonists) can release histamine, and they have been shown to lead to arrhythmias (Llenas et al., 1999). These arrhythmias are usually explained by the inhibitory action of these drugs on potassium channels leading to prolonged duration of the AP, which are delayed after-depolarisations to *torsade de pointes* arrhythmias (Llenas et al., 1999). Other researchers have argued that both compounds can accumulate in the heart and release histamine, which stimulates H_2_-histamine receptors and thus elicits arrhythmias (Llenas et al., 1999).

### 11.2 Roles of Histamine and Histamine Receptors in Ischaemia and Hypoxia

There is some evidence that in a minority of patients with Prinzmetal-angina, a form of angina pectoris in which coronary arteries contract despite histologically normal endothelial and smooth muscle cells, the causative agent might be histamine. In these patients, it has been speculated that their coronary arteries are less susceptible to H_2_R-mediated vasodilatation and prone to H_1_R-mediated vasoconstriction, which was explained by a higher density of mast cells near the coronary arteries, altered function of mast cells that facilitated the release of histamine and/or deleterious alterations in endothelial cells, including less histamine receptor mediated vasodilatation caused by blocked signal transduction in them (Ginsburg et al., 1981; Okumura et al., 1991). It is well known that cardiac ischaemia leads to the release of adenosine, which is thought to dampen the effect of adrenaline and which might be regarded as an anti-adrenergic effect of adenosine. Interestingly, an “anti-histaminergic” effect of adenosine has been noted. Adenosine has been reported to inhibit the stimulatory effect of histamine (via H_2_-histamine receptors) on adenylyl cyclase activity (Endoh 1979; Baumann et al., 1981a). Moreover, the interaction of isoprenaline and histamine has been reported, in which histamine reduced the β-adrenoceptor-mediated increase in L-type Ca^2+^ current in guinea pig ventricular cardiomyocytes (Belevych et al., 2004). An ischaemia-mediated release of noradrenaline from the heart was attenuated by histamine acting on H_4_-histamine receptors in cardiac ganglia (Aldi et al., 2014) as well via H_3_-histamine receptors, as previously mentioned (Koyama et al., 2003b). In patients, an acute myocardial infarction was accompanied by an increase in the plasma histamine levels, which was reported in reviews by Reid et al. (2011) and Luo et al. (2013). In animal hearts, the release of histamine by ischaemia was described in early research (Genovese et al., 1988).

Ischaemia and reperfusion led to detrimental increases in the permeability of the endothelial layers of arterial vessels mediated by H_1_-histamine receptors and impaired the function of mitochondria in cardiomyocytes. These detrimental events partially resulted from the activation of H_2_-histamine receptors by histamine released from cardiac mast cells in reperfusion. This hypothesis is supported by the fact that in mice pre-treated with famotidine or with general KO of the H_2_R, ischaemia alone (24 h occlusion of left coronary artery) or ischaemia (1 h occlusion of left coronary arteries) and reperfusion (24 h) led to less myocardial necrosis and thus to less inhibition of cardiac function than in WT hearts (Luo et al., 2013). However, these studies were mainly performed in neonatal rat cardiomyocytes that contained inotropically active H_2_-histamine receptors, whereas these receptors are inactive in adult mouse cardiomyocytes and hearts (Gergs et al., 2019). Hence, it could be hypothesised that the beneficial results of H_2_R KO or famotidine treatment are due to the lack or blockade of H_2_-histamine receptors in non-muscle cells of the heart, such as fibroblasts, endothelial cells, smooth muscle cells and mast cells (Table 5). In contrast, isolated left atrial preparations of mice that overexpressed H_2_R in the heart showed greater resilience against hypoxia compared with the WT control preparations (Gergs et al., 2020). However, in the isolated left ventricle with global ischaemia, preparations from mice that overexpressed H_2_R in the heart showed a more rapid decline in force under these ischaemic conditions compared with WT control preparations (Gergs et al., 2020). Thus, the protective or deleterious effects of H_2_-histamine receptors might be dependent on the region of the mammalian heart. Further research should be conducted to investigate whether these regional differences are also present in the human heart.

## 12 Roles of Histamine and Histamine Receptors in Chronic Heart Failure

Currently, the involvement of histamine and its receptors in the genesis, maintenance and prevention of chronic heart failure is insufficiently understood. The following sections give an overview of the possible implications of histamine and histamine receptors for different kinds of heart failure in animal models and in humans. To illustrate, Figures 3, 5 show potentially involved signalling pathways.

### 12.1 Animal Models of Chronic Heart Failure

#### 12.1.1 Ischaemia-Induced Heart Failure

In guinea pigs, where heart failure was induced by closing a coronary artery, the positive inotropic effect of β-adrenoceptor agonists was blunted; however, histamine showed a positive inotropic effect (Baumann et al., 1982). These findings are in line with results of samples drawn from human hearts, where the efficacy of histamine in increasing the force of contraction was preserved in patients with end-stage heart failure, which is discussed in the following sub-section.

#### 13.1.2 Pressure- or Volume-Induced Heart Failure

Using transverse aortic constriction, mice pre-treated with famotidine or lacking H_2_-histamine receptors (H_2_R KO mice) showed better cardiac performance and less histological damage compared with WT mice (Zeng et al., 2014). These results were explained by H_2_R-induced cardiac fibrosis and apoptosis in WT mice. In addition, the researchers used neonatal rat cardiomyocytes and fibroblasts. The results showed that the activation of H_2_-histamine receptors led to increased apoptosis of cardiomyocytes and fibrosis via fibroblast activation (Zeng et al., 2014). However, as adult rats and adult mice show no inotropically active H_2_-histamine receptors, this finding is difficult to understand (Zeng et al., 2014; Gergs et al., 2019). Perhaps the lack of H_2_-histamine receptors in the fibroblasts of KO mice could partially explain these findings.

In a guinea pig model of heart failure as a result of a pressure overload by infusion of vasopressin, H_2_R agonists such as impromidine exhibited a positive inotropic effect and a positive chronotropic effect (Felix et al., 1991b). This result was interpreted as indicating that H_2_R-stimulated inotropic pathways were still active in chronic heart failure, which was in line with findings in humans (Felix et al., 1991b).

In dogs, heart failure induced by volume overload due to surgically induced mitral insufficiency, an increased density of cardiac mast cells was observed (Stewart et al., 2003). Subsequently, in a rat model of heart failure, namely volume overload by surgically producing a hemodynamically relevant fistula in the abdomen of rats, nedocromil, a mast cell stabiliser that mitigated the release of histamine from mast cells, reduced mechanical dysfunction, cardiac hypertrophy, and the combined end points of morbidity and mortality (Brower and Janicki, 2005). In this model system, mast-cell-deficient rats showed less impairment of cardiac function under volume overload (Levick et al., 2008). Mast cells contain histamine, which was increased in this model of heart failure. This finding was suggested to be in line with findings in human chronic heart failure patients where mast cell density and histamine content were found to increase and could be interpreted as proof of the principle that volume overload in patients alters cardiac histamine content.

Similarly, in pressure-induced heart failure in spontaneously hypertensive (SHR) rats, an increase in cardiac histamine levels and an increase in the density of H_2_-histamine receptors were observed (Potnuri et al., 2018). However, as previously (Interactions Between Histamine, Histamine Receptors, and Noradrenaline), a conceptual problem is that histamine in rat heart acts on the force of contraction not via H_2_R but via the release of endogenous catecholamines (Laher and McNeill 1980a). Hence, additional actions of histamine must be operational here. Famotidine improved systolic and diastolic function in SHR, reduced cardiac hypertrophy, reduced cardiac fibrosis, reduced histamine concentrations, elevated calcineurin activity, and the phosphorylation of protein kinase B (AKT) in SHR compared with the controls (Potnuri et al., 2018). These effects were explained as follows: famotidine might inhibit mast cell degranulation by blocking H_2_-histamine receptors on the mast cells (Potnuri et al., 2018).

#### 12.1.3 Drug-Induced Heart Failure

Doxorubicin is well known to induce chronic heart failure in humans. The mechanism by which it occurs is still disputed. In a dog model, the application of doxorubicin in concentrations that led to heart failure also increased cardiac histamine levels. The authors speculated that this mechanism might come into play in human patients (Bristow et al., 1981). Similar findings were reported in rats treated with doxorubicin, which led to elevated levels of histamine in the isolated right atria (Decorti et al., 1997).

#### 12.1.4 Myocarditis-Induced Heart Failure

Rats were injected with a preparation containing porcine myosin as an antigen, which over time led to myocarditis. Ranitidine did not reduce the loss of cardiac contractility due to myocarditis, whereas a H_4_R antagonist was beneficial (Stasiak et al., 2018). These results indicate that targeting H_2_R is not generally beneficial in the treatment of chronic heart failure. Furthermore, these data indicate that H_2_R antagonists in general would not be useful in autoimmune myocarditis and resultant heart failure. However, to the best of our knowledge, comparable human data are currently lacking. Myocarditis due to the encephalo-myocarditis virus was more pronounced in WT hearts than in two lines of mast-cell-deficient mice. The cardiac function of these mice was improved by administering an H_1_R antagonist (Higuchi et al., 2008).

#### 12.1.5 Tachycardia-Induced Heart Failure

In a dog model of tachycardia-induced heart failure, pacemakers were implanted, and the hearts were stimulated at high beating rates for a prolonged period, which eventually led to heart failure. Samples drawn from canine hearts showed increasing densities of cardiac mast cells and elevated cardiac levels of histamine in a time-dependent manner (Takahama et al., 2010).

#### 12.1.6 Genetically Induced Heart Failure as a Model System

Preliminary data suggest that under certain conditions, H_2_R may be beneficial in treating cardiac hypertrophy and failure. In a genetic model of cardiomyopathy and contractile dysfunction in mice that overexpressed the catalytic subunit of the serine/threonine protein phosphatase 2A (PP2A) to the heart, crossbreeding with mice that overexpressed human H_2_R, improved cardiac function (Gergs et al., 2020).

### 12.2 Human Heart Failure

#### 12.2.1 Heart Failure and Histamine

It has been suggested that mastocytosis caused by increasing histamine levels might contribute to the development of heart failure (Klock et al., 2007). In the blood of patients with a special subtype of chronic heart failure, namely idiopathic dilative cardiomyopathy (IDC), histamine levels were increased (Zdravkovic et al., 2011). This elevated histamine has been speculated to be fibrinogenic, which could contribute to cardiac fibrosis observed in heart failure (Patella et al., 1998). Hence, there could be a positive feedback loop between histamine levels in the heart and a positive inotropic effect of histamine in the human heart. However, the activity of DAO, a histamine degrading enzyme, was increased in patients with heart failure, which was reported in a review by Stolen et al. (2004). Hence, it could be speculated that the increase in DAO is used as a counterbalance to protect the heart against excessively high plasma histamine levels.

#### 12.2.2 Heart Failure and H_2_R Agonists

The positive inotropic effect of histamine was observed in samples from patients in which the positive inotropic effect of β_1_-adrenoceptor stimulation was diminished (Bristow et al., 1982a). Similarly, the histamine maintained the ability to increase the activity of adenylyl cyclase in failing human heart samples, in which the coupling of noradrenaline with the activity of adenylyl cyclase was attenuated (Bristow et al., 1982a; 1982b). However, as mentioned above, histamine is not a useful inotrope; it also acts on all other histamine receptors, and to a large extent, it is metabolised and thus inactivated if taken orally by chronic heart failure patients. Hence, it is important to find and test H_2_R selective agonists (Table 1). One H_2_R selective agonist was found in the form of impromidine (Table 1). It was found to be active as a positive inotropic agent in heart failure patients. In patients with end-stage congestive heart failure and intact coronary blood flow, the force of cardiac contraction could no longer be increased by the stimulation of β-adrenoceptor agonists using dobutamine, which, clinically, is often called “catecholamine refractory heart failure”. However, in severely ill patients, impromidine increased cardiac output, decreased pulmonary capillary wedge pressure and decreased systemic vascular resistance (Baumann et al., 1984; Felix et al., 1995). Impromidine was not tested further because the authors noted increases in gastric acid secretion, which was caused by H_2_R agonists in the stomach, and in cardiac arrhythmias, which are commonly found in connection with cAMP-elevating agents (Felix et al., 1995). The same research group argued that because the concentration response curve was bell-shaped, the H_2_R-mediated increase in gastric secretion in impromidine-treated patients might be self-limiting and that the patients always complained about a skin flush that was accounted for by cutaneous vasodilation (Baumann et al., 1984).

In line with the positive inotropic effect of H_2_R agonists in human heart failure, the density of H_2_-histamine receptors was unaltered in chronic heart failure patients, whereas in the same human cardiac samples, the density of β_1_-adrenoceptors was diminished (Baumann et al., 1984). It is unclear whether the preserved ability of histamine to generate cAMP in failing human hearts is really beneficial. It has been speculated that the histamine-induced cAMP increase in failing hearts might, in part, explain deadly cardiac arrhythmias in these patients, as cAMP is known to increase the propensity to generate arrhythmias presumably by increasing Ca^2+^ influx into heart cells (Leary et al., 2018b).

Here, a further caveat is in order. Another research group noted that the positive inotropic effect of histamine in failing human cardiac ventricular trabeculae was diminished (Brown et al., 1986; Böhm et al., 1988b). Whether this was the result of different techniques of contraction measurement, different pre-operative drug therapy, or different patient characteristics was never resolved. However, it is a clinically relevant discrepancy in the field that should be addressed in future research.

#### 12.2.3 H_2_R Antagonists in Heart Failure

Registered data on Japanese patients showed that the administration of the H_2_R antagonist famotidine reduced the incidence of heart failure (Kim et al., 2004). Similar results were observed in a clinical study where the incidence of cardiac remodelling in heart failure patients decreased with famotidine treatment (Kim et al., 2006). Of major interest in our context is a 10-years progressive observational study on initial non-heart disease patients. In this study, H_2_R antagonists such as famotidine reduced the development of not only left ventricular hypertrophy (Leary et al., 2016) but also right ventricular hypertrophy (Leary et al., 2014). Another cause of right-sided heart failure is pulmonary hypertension, a disease with high mortality. The registered data suggest that pulmonary hypertensive patients who were administered famotidine had lower mortality (Leary et al., 2018b). A nationwide Danish registry study compared new users of proton pump inhibitors or H_2_R antagonists after a hospital stay because of heart failure. The rate of hospital admissions for worsening heart failure and one- and 5-years total mortality were lower in H_2_R antagonist-treated patients (Adelborg et al., 2018). The question has been raised whether famotidine is the best choice of an H_2_R antagonist for the treatment of heart failure. Unlike burimamide, famotidine is not a pure antagonist but an inverse agonist or a biased agonist (Alonso et al., 2015; Leary et al., 2016).

What causes the beneficial effects of famotidine? It has been suggested that famotidine acts on mast cells and not on cardiomyocytes (Klock et al., 2007). Correspondingly, the density of mast cells is higher in patients with heart failure (Patella et al., 1998). Others have speculated that famotidine has an indirect effect by blocking H_2_-histamine receptors; thus, cardiac histamine is free to act on H_3_-histamine receptors. Histamine is more potent on H_3_-histamine receptors than on H_2_-histamine receptors (Panula et al., 2015). Thus, via H_3_R, cardiac histamine might reduce the release of noradrenaline from cardiac ganglia (Panula et al., 2015), thus potentially abrogating the deleterious effects of noradrenaline on cardiac β-adrenoceptors (Asanuma et al., 2006). The unhindered stimulation of β-adrenoceptors can lead to cardiac hypertrophy (e.g., Gergs et al., 2020). In patients with coronary heart disease, the vasoconstrictory effects of histamine on arteriosclerotic vessels have been speculated to be reduced by famotidine (Ginsburg et al., 1981; Asanuma et al., 2006). Others have speculated that the beneficial effects might result from altered renal blood flow, reduced systemic blood pressure or the reduced detrimental remodelling of the heart due to the action on fibroblasts (Leary et al., 2014). However, the present review revealed conflicting results. Some researchers noted increased mortality from heart failure in patients treated with famotidine (Yoshihisa et al., 2017). Thus, timing, duration and dose of famotidine or subtle differences in the clinical characteristics of studies may account for conflicting results. Thus, further clinical studies on famotidine in cardiac hypertrophy are necessary to improve the stratification of patients.

#### 12.2.4 Mutations of Histamine Receptors and Human Heart Failure

A study on Han Chinese showed a correlation between a mutation of H_3_R but not of H_2_R, DAO, or histamine N-methyl-transferase (HMT) and the risk of developing systolic heart failure (He et al., 2016). A recent study, which seems to be the only one to connect mutations of H_2_-histamine receptors and heart failure, reported four relevant single nucleotide polymorphisms in the deoxyribonucleic acid (DNA) extracted from peripheral leukocytes in participants. The allele rs2241562 was significantly correlated with chronic heart failure in US patients with a Chinese heritage and a history of hypertension (Leary et al., 2018a). The allele rs2241562 is an intron variant, and it may be relevant for the stability of the RNA, or it may be a transcription enhancer. This allele was present only in the participants of Chinese heritage and not in participants of other ethnicities who took part in this trial (Leary et al., 2018a). The same report included a different population of patients with systolic heart failure due to idiopathic cardiomyopathy at the time of randomisation (Leary et al., 2018a). Heart failure was defined as a left ventricular ejection fraction lower than 40% using ventriculography (Leary et al., 2018a). The study participants were treated with the β-adrenoceptor antagonists carvedilol or metoprolol. The participants underwent a biopsy in the right ventricular distal septum before and after treatment with β-adrenoceptor antagonists (Leary et al., 2018a). From these biopsies, mRNA was isolated and sequenced (Leary et al., 2018a). Two transcript variants of human H_2_R were identified in coding for proteins comprised of 397 or 359 amino acids (Leary et al., 2018a). The shorter 359 amino acid variant was found to be homologous to the originally cloned human H2R and was designated as the canonical variant (Leary et al., 2018a). Hence, this study has shown the actual presence of different messenger RNAs of H_2_-histamine receptors in the human right ventricle (Leary et al., 2018a). Participants who responded to β_1_-adrenoceptor antagonist treatment with an increase in the left ventricular ejection fraction by more or equal to 10 absolute percentages (in this study called super-responders) had a higher expression of mRNA coding for the shorter (359 amino acids) protein isoform of H_2_R (Leary et al., 2018a). In contrast, participants who did not improve their left ventricular ejection fraction under therapy with β-adrenoceptor antagonists, the so-called non-responders, exhibited a lower expression of the mRNA coding of the longer variant (397 amino acids) as well as a lower expression of the summary of both variants of H_2_R in right ventricular biopsies (Leary et al., 2018a). Whether these two receptor variants displayed a differential function profile is not yet known; moreover, the studied cohorts were small. In other words, it remains to be elucidated whether such changes in the expression of variants of H_2_R in the human heart contribute to the success of β-adrenoceptor antagonist therapy and whether this information could be used to improve patient stratification and treatment. However, the researchers recommended further research on H_2_-histamine receptors and their role in human heart failure.

## 13 Sepsis and Acute Heart Failure

Over decades of research, there has been consensus that in septic shock, histamine levels in plasma increase. An example is a hundred-fold increase in the plasma of rabbits, as reported in a review by Matsuda et al. (2002). In a rat model of septic shock, the mortality of the animals was lowered by administering both H_1_R and H_2_R antagonists (Brackett et al., 1985). In HDC KO mice, the injection of lipopolysaccharide (LPS), a model of sepsis, in living mice led to lower increases in IL-6 in serum or liver (heart was not reported) than in WT (Horvath et al., 2002). In septic mice subjected to LPS treatment to induce sepsis, the prior application of a drug (amodaiquine) that inhibits the activity of histamine-methyl transferase (an enzyme that inactivates histamine), increased tissue levels of histamine in the liver, and reduced sepsis-induced mortality in mice, which was explained, in part, by the measured reduction in the tumour necrosis factor alpha (Yokoyama et al., 2007). In a rabbit model of sepsis, sepsis-induced tachycardia was blunted by a H_2_R antagonist (Matsuda et al., 2002). Sepsis increased mRNA levels of H_2_- and H_1_- histamine receptors in the atrium and ventricle of septic rabbits (Matsuda et al., 2002). If lipopolysaccharides were used to induce sepsis in mice, the role of histamine and H_2_-histamine receptors was corroborated. In H_2_R KO mice and HDC KO mice, sepsis was more lethal than in WT mice (Yokoyama et al., 2004). The beneficial effects of H_2_R stimulation have been explained by the fact that in H_2_R null mice, LPS injection in the animals led to higher levels of cytokines and histologically confirmed liver damage. However, the heart was not examined (Masaki et al., 2005). In isolated human monocytes, LPS increased the expression of adhesion molecules, which was mediated by H_2_-histamine receptors (Morichika et al., 2003). This result led to the suggestion that sepsis therapy could be improved by the application of H_2_R antagonists (Takahashi et al., 2004). Similarly, mice with cardiac overexpression of H_2_R were more susceptible to the detrimental effect of LPS in the left ventricular ejection fraction compared with littermate WT control mice (Gergs et al., 2020).

## 14 Cardiovascular H_2_-Histamine Receptors and Ageing

The data on this relationship are limited; hence, further research is warranted. The H_2_R-induced relaxation of isolated aortic strips using dimaprit as an agonist was greatly attenuated in mature rabbits (6–7 months of age) compared to young rabbits (6 weeks of age) (Holl and Mokler 1982). A contrasting finding was reported in strips isolated from coronary arteries in dogs. In older beagle dogs (2 years and 12 years), H_2_R-induced relaxation was more potent and effective than in young beagle dogs (80–110 days of age) (Toda et al., 1987). Neonatal and adult animal models are discussed in *Age-Dependent Histamine Effects*.

In human subjects aged from 20 to 81 years, the vasodilatory effects of histamine via H_2_-histamine receptors diminished with increasing age, while the vasodilatory effects of H_1_-histamine receptors did not change during aging (Bedarida et al., 1995). In this study, the effects of the intravenous infusion of histamine (2–136 ng histamine/min in the absence or presence of 49 μg/min of the H_2_R antagonist cimetidine or 530 ng/min of the H_1_R antagonist brompheniramine on the diameter of the dorsal hand veins) were studied (Bedarida et al., 1995).

## 15 Cardiovascular H_2_-Histamine Receptors and Exercise

In healthy male subjects undergoing exercise by cycling, an increase in plasma histamine levels was observed (Doh et al., 2016). After a longer duration (more than 15 min) of skeletal muscle exercise, both H_1_- and H_2_-histamine receptors mediated post-exercise hyperaemia (Doh et al., 2016). Post-exercise systemic vascular pressure was reduced in both men and women, and these reductions were attenuated when 300 mg ranitidine (Table 2) per os or a combination of 540 mg fexofenadine and 300 mg ranitidine per os were administered (McCord et al., 2006a; McCord and Halliwill, 2006b). The beneficial effects of H_1_R and H_2_R antagonism were accompanied by and conceivably mediated by an increase in skeletal muscle perfusion in humans (Van der Stede et al., 2021). In patients with high normal hypertension (systolic blood pressure 120–139 mmHg in males aged 20–27 years), the effects of the blockade of H_1_- and H_2_-histamine receptors on post-exercise hemodynamics were lower than in normotensive subjects (Naylor et al., 2020). These results suggested that under pathological conditions (higher blood pressure), the vasodilatory effects of H_1_-histamine receptors on endothelial cells and of H_2_-histamine receptors on smooth muscle cells in the vessels of the skeletal musculature might be blunted (Naylor et al., 2020). Another interpretation of these data might be that hypertension is in part due to the functional impairment of H_2_- and H_1_-histamine receptors.

## 16 Outlook

From a mechanistic point of view, a real (not only virtual) crystal structure of human H_2_R at a good spatial resolution is crucial to better understand the receptor. The next logical step is the generation of crystal structures using histamine or dimaprit. Thereafter, crystal structures with binding proteins, such as stimulatory or inhibitory GTP-binding proteins and other signal transduction proteins, would be important. The subcellular localisation of human H_2_R should be studied in much more detail. It might not be confined to the sarcolemma, which would have functional implications that are still unknown. The improved knowledge of the regulation of the promoter of human H_2_R should be another research goal. Another important step forward involves the production and characterisation of a reliable antibody for detecting human H_2_R in Western blots. This antibody would enable research on diseases that alter the expression of human H_2_R on the protein level, such as ischaemia, which would enable the development of a therapeutic intervention. Another step is the development of cell-type specific agonists and antagonists of human H_2_R, which might be achieved by the typical synthesis of new small molecules. Alternatively, a virus that has a cell type-specific promoter could be developed to code receptors. Novel cell type-specific agonists might make it possible, for example, to increase the force of contraction without acting on the sinus node. In other words, a positive inotropic effect that did not require high oxygen expenditure by simultaneously increasing the beating rate might be achieved by these novel compounds. Conversely, using smooth muscle specific H_2_R agonists, blood pressure could be reduced without increasing the force of contraction. An open question remains regarding the role of histamine in arrhythmogenesis in humans. Finally, although they would be expensive, clinical trials conducted to test the usefulness of H_2_R therapy in treating various forms of congestive heart failure would contribute to not only the literature but also the efficacious treatment of patients with this disease.

## Glossary

**5-HT**_**4**_ **receptor**: serotonin 4 receptor
β-MHC: beta myosin heavy chain
AKT: protein kinase B
ANP: atrial natriuretic peptide(s)
AP: action potential
APD: action potential duration
ATP: adenosine triphosphate
AV: atrioventricular node
Bax: homolog of Bcl-2, an apoptosis activator
CaMKII: Ca^2+^ and calmodulin-dependent protein kinase
cAMP: 3′, 5′- cyclic adenosine monophosphate
cGMP: 3′, 5′- cyclic guanosine monophosphate
CHO: Chinese hamster ovary cells
CREB: cAMP-dependent transcription factor
COX2: Cyclooxygenase 2
CSQ: calsequestrin
DAD: delayed action potential
DAG: diacylglycerol
DAO: diamine oxidase
DAPK2: death-associated protein kinase 2
DNA: deoxyribonucleic acid
EC: endothelial cells
**EC**_**50**_ **-value**: half maximal effective concentration values
ECG: electrocardiography
EHNA: erythro-9-(2-hydroxy-3-nonyl)adenine
ERK1/2: extracellular regulated receptor kinase 1/2
G-protein: guanosine triphosphate (GTP) binding protein
GC: guanylyl cyclase
**GDP-**_**ϒ**_ **-S**: in gamma position sulphur substituted guanosine triphosphate
GIRK: GTP-binding protein coupled inwardly rectifying potassium current
GRK: G-protein coupled receptor kinase 2
**H**_**2**_ **-TG**: transgenic mice with heart-specific overexpression of the H_2_-histamine-receptor
**H**_**1**_ **R**: H_1_-histamine receptor
**H**_**2**_ **R**: H_2_-histamine receptor
HCN: I_f_-currents, hyperpolarisation-activated ion channel
HDC: histidine decarboxylase
HIF: hypoxia-inducible transcription factors
HMT: histamine N-methyl-transferase
IDC: idiopathic dilative cardiomyopathy
I(f): the hyperpolarisation-activated inward current (formerly known as I(h)
**I**_**K. Ach**_: G-protein gated potassium channel
**I**_**Kr**_: rapid delayed rectifier potassium current
**I**_**Ks**_: slow delayed rectifier potassium current
Indo-1: calcium indicator
**IP**_**3**_: inositol trisphosphate
JNK: c-jun N terminal kinase
KO: knock out: deletion of a gene in a mouse
LPS: lipopolysaccharide, a model of sepsis
LTCC: L-type Ca^2+^ channel
MAPK: mitogen activated protein kinase
NCE: negative chronotropic effect
NCX: sodium/calcium exchanger
NE: noradrenaline (=norepinephrine)
NFAT: nuclear factor of activated T-cells
NIE: negative inotropic effect
NO: nitric oxide
NOS: nitric oxide synthase
P38: p38 mitogen activated protein kinase
PCE: positive chronotropic effect
PDE: phosphodiesterase
PEA: 2-pyridylethylamine, H_1_R agonist
PIE: positive inotropic effect
**PLA**_**2**_: phospholipase A_2_
PLC: phospholipase C
PKC: protein kinase C
PLB: phospholamban
PP1: serine threonine protein phosphatase 1
PP2A: serine threonine protein phosphatase 2A
RNA: ribonucleic acid
RYR: ryanodine receptor
SA: Sinus node
SERCA: SR-Ca^2+^-ATPase
SHR: spontaneously hypertensive rats
SMA: smooth muscle actin
SMC: smooth muscle cells
SR: sarcoplasmic reticulum
ThEA: 2-(2-thiazolyl)-ethylamine, a H1R agonist
TnI: inhibitory subunit of troponin
TnFα: tumour necrosis factor alpha
WT: wild type
**V**_**max**_: maximal velocity of the action potential
